# Elafin Reverses Intestinal Fibrosis by Inhibiting Cathepsin S-Mediated Protease-Activated Receptor 2

**DOI:** 10.1016/j.jcmgh.2022.06.011

**Published:** 2022-07-14

**Authors:** Ying Xie, Lindsey Fontenot, Andrea Chupina Estrada, Becca Nelson, Jiani Wang, David Q. Shih, Wendy Ho, S. Anjani Mattai, Florian Rieder, Dane D. Jensen, Nigel W. Bunnett, Hon Wai Koon

**Affiliations:** 1 Vatche and Tamar Manoukian Division of Digestive Diseases, David Geffen School of Medicine at the University of California Los Angeles, Los Angeles, California; 2 Department of Gastroenterology, The First Hospital of China Medical University, Shenyang City, Liaoning Province, China; 3 F. Widjaja Foundation, Inflammatory Bowel & Immunobiology Research Institute, Cedars-Sinai Medical Center, Los Angeles, California; 4 Department of Medicine, David Geffen School of Medicine at the University of California Los Angeles, Los Angeles, California; 5 Department of Gastroenterology, Hepatology, and Nutrition, Digestive Diseases and Surgery Institute, Cleveland Clinic Foundation, Cleveland, Ohio; 6 Department of Molecular Pathobiology, Department of Neuroscience and Physiology, Neuroscience Institute, New York University, New York, New York; 7 Bluestone Center for Clinical Research, New York University College of Dentistry, New York, New York

**Keywords:** Fibrosis, Protease, Receptor, ANOVA, analysis of variance, CD, Crohn’s disease, CD-HIF, Crohn’s disease patient-derived primary intestinal fibroblasts, CDSE, Crohn’s disease patients’ serum exosomes, COL1A2, collagen type I alpha 2, CTSS/Ctss, gene name of cathepsin S or CatS, HPMC, hydroxypropyl methylcellulose, IBD, inflammatory bowel disease, ODA, overall disease activity, PAR1/2, protease-activating receptor 1/2, PMBC, peripheral blood mononuclear cell, SLPI, secretory leukocyte protease inhibitor, TGF-β1, transforming growth factor beta 1, TNBS, trinitrobenzene sulfonic acid, TNF, tumor necrosis factor, ZEB1, zinc finger E-box-binding homeobox 1

## Abstract

**Background & Aims:**

More than half of Crohn’s disease patients develop intestinal fibrosis-induced intestinal strictures. Elafin is a human protease inhibitor that is down-regulated in the stricturing intestine of Crohn’s disease patients. We investigated the efficacy of elafin in reversing intestinal fibrosis and elucidated its mechanism of action.

**Methods:**

We developed a new method to mimic a stricturing Crohn’s disease environment and induce fibrogenesis using stricturing Crohn’s disease patient-derived serum exosomes to condition fresh human intestinal tissues and primary stricturing Crohn’s disease patient-derived intestinal fibroblasts. Three mouse models of intestinal fibrosis, including SAMP1/YitFc mice, *Salmonella*-infected mice, and trinitrobenzene sulfonic acid–treated mice, were also studied. Elafin-Eudragit FS30D formulation and elafin-overexpressing construct and lentivirus were used.

**Results:**

Elafin reversed collagen synthesis in human intestinal tissues and fibroblasts pretreated with Crohn’s disease patient-derived serum exosomes. Proteome arrays identified cathepsin S as a novel fibroblast-derived pro-fibrogenic protease. Elafin directly suppressed cathepsin S activity to inhibit protease-activated receptor 2 activity and Zinc finger E-box-binding homeobox 1 expression, leading to reduced collagen expression in intestinal fibroblasts. Elafin overexpression reversed ileal fibrosis in SAMP1/YitFc mice, cecal fibrosis in *Salmonella*-infected mice, and colonic fibrosis in trinitrobenzene sulfonic acid–treated mice. Cathepsin S, protease-activated receptor 2 agonist, and zinc finger E-box-binding homeobox 1 overexpression abolished the anti-fibrogenic effect of elafin in fibroblasts and all 3 mouse models of intestinal fibrosis. Oral elafin-Eudragit FS30D treatment abolished colonic fibrosis in trinitrobenzene sulfonic acid–treated mice.

**Conclusions:**

Elafin suppresses collagen synthesis in intestinal fibroblasts via cathepsin S-dependent protease-activated receptor 2 inhibition and decreases zinc finger E-box-binding homeobox 1 expression. The reduced collagen synthesis leads to the reversal of intestinal fibrosis. Thus, modified elafin may be a therapeutic approach for intestinal fibrosis.

## Graphical abstract

**Figure:**
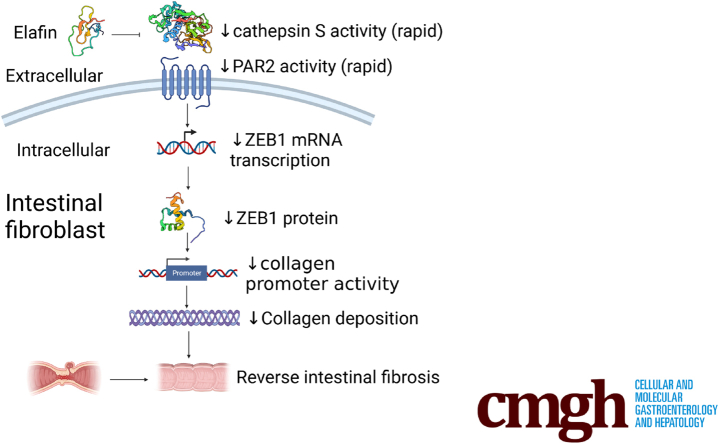

**Summary**

Elafin inhibits cathepsin S and protease-activated receptor 2 activities and reduces collagen synthesis in intestinal fibroblasts.

Intestinal stricture is a debilitating complication of inflammatory bowel disease (IBD).^1^ Approximately 30%–50% of Crohn’s disease (CD) patients develop intestinal strictures.^2^^,^^3^ Anti–tumor necrosis factor (TNF) alpha neutralizing antibodies fail to reverse intestinal strictures in CD patients.^4^ Strictureplasty can alleviate mild and simple bowel narrowing, but surgical resection may be needed to resolve severe and complex bowel obstructions.^5^ However, surgery may adversely affect the patients’ quality of life. Thus, new therapeutic approaches to intestinal fibrosis are needed.

To discover stricture-related targets, we compared colonic mRNA expression in stricturing and non-stricturing CD patients using whole-transcriptome RNA sequencing.^6^ Consistent with a similar study,^4^ stricturing CD patients have increased fibrosis-associated extracellular matrix gene and transcriptional regulator expression. However, we noted that these patients had impaired epithelial gene expression such as keratin, mucin, and antimicrobial peptide (elafin).^6^

IBD patients also carry specific patterns of microRNAs, proteins, and lipids in serum exosomes.^7^ Exosomes are small vesicles in blood and body fluids.^8^ Treatment of human intestinal fibroblasts with serum exosomes from stricturing CD patients (CDSE) caused increased mRNA expression of collagen type I alpha 2 (COL1A2) and alpha-smooth muscle actin.^6^ Components in serum exosomes can regulate intestinal cell functions.^9^ Although the pro-fibrogenic mechanism of CDSE is not fully understood, CDSE has lower miR205 expression than serum exosomes in non-stricturing CD patients.^6^ The miR205 possesses anti-fibrogenic properties as its inhibition increases collagen expression in intestinal fibroblasts.^6^ Collagen is a prominent extracellular matrix component in the CD intestinal strictures.^4^^,^^6^ Therefore, CDSE treatment of intestinal fibroblasts can partially mimic a stricturing CD environment in vitro.

An antimicrobial peptide (cathelicidin) possesses anti-fibrogenic effects^10^ but is cytotoxic.^11^ Another human antimicrobial peptide (elafin) functions as a protease inhibitor.^12^^,^^13^ IBD patients have increased elafin protein levels in circulation^6^ but reduced elafin mRNA expression in the peripheral blood leukocytes.^14^^,^^15^ Patients with ulcerative colitis have increased colonic elafin mRNA expression.^6^^,^^16^ Several groups reported different colonic elafin mRNA and protein expression in CD patients.^6^^,^^17^^,^^18^ Interestingly, stricturing CD patients have lower colonic elafin expression than non-stricturing CD patients.^6^

Elafin may also be used for therapeutic purposes. Elafin protects intestinal barrier function by inhibiting elastase activity.^19^ Adenoviral delivery of elafin ameliorated chemically induced colitis.^20^ In mice, oral administration of elafin-expressing *Lactococcus* inhibited colitis and gluten-related disorders.^8^^,^^21^ Oral elafin formulation was as efficacious as lentiviral elafin overexpression in reversing obesity and diabetes in high-fat diet–treated mice.^22^ Intravenous infusion of elafin to increase circulating levels (4.5 μg/mL) is also safe without affecting plasma elastase activity and cytokine levels in patients.^23^ Therefore, elafin is an attractive therapeutic target against intestinal inflammation and fibrosis.

Elafin, an antiprotease, may be helpful against intestinal fibrosis because proteases participate in fibrogenesis. For example, tryptase can induce collagen expression in human colonic fibroblasts,^24^ whereas *Salmonella*-infected mice have increased protease expression in fibrotic cecal tissues.^25^ These previous studies provided a premise to explore whether elafin can inhibit fibrogenesis in intestinal fibroblasts via a specific antiprotease mechanism, leading to the amelioration of intestinal fibrosis.

This study discovered the functions of novel mediators of fibrogenesis. We elucidated a new anti-fibrogenic mechanism of elafin using primary stricturing CD patient-derived intestinal fibroblasts (CD-HIF), primary human colonic epithelial cells, Crohn’s disease patient-derived peripheral blood mononuclear cells (PBMCs), fresh human intestinal tissues, and 3 CD-relevant mouse models of intestinal fibrosis, including well-established chronic trinitrobenzene sulfonic acid (TNBS)–mediated colitis,^26^ *Salmonella*-infected mice with cecal Th1/Th17 cytokine activation,^27^ and CD-like SAMP1/YitFc mice.^28^^,^^29^ We also used an orally active elafin formulation to determine the feasibility of oral elafin therapy against intestinal fibrosis.

## Results

### Elafin Inhibited Collagen Synthesis in Activated Human Colonic Tissues and Fibroblasts

**Figure 1:**
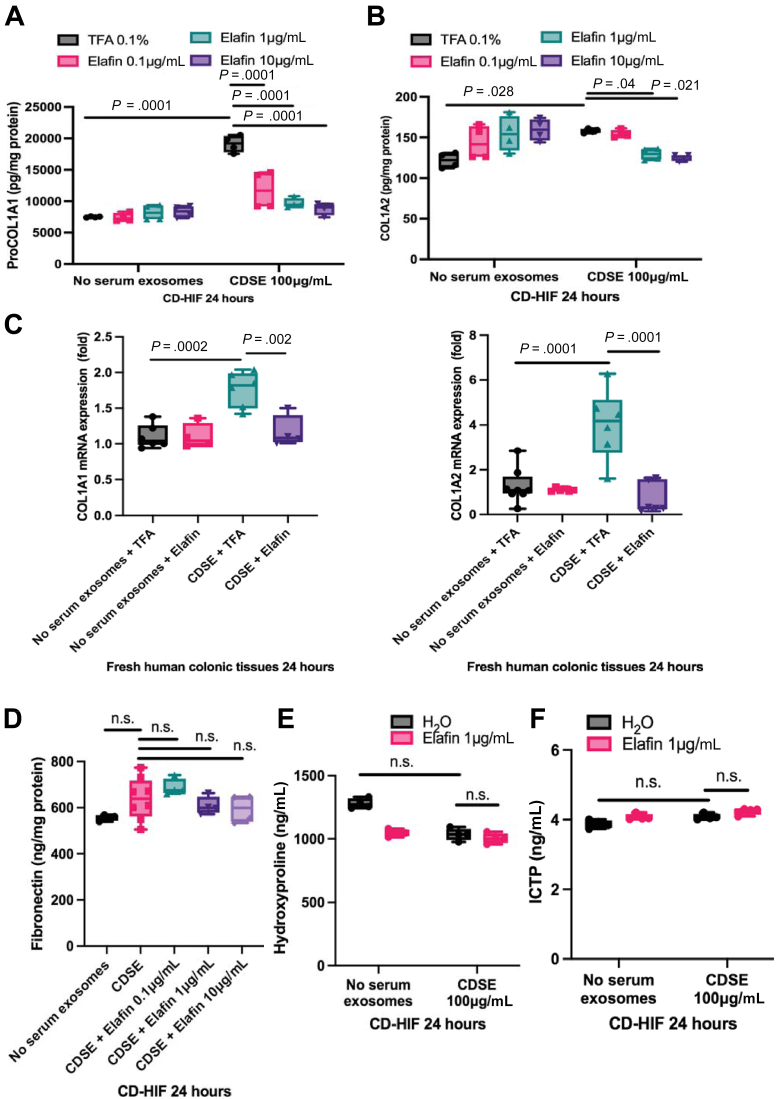
**(*A* and *B*) Primary stricturing CD patient-derived intestinal fibroblasts (CD-HIF) were pretreated with or without 100 μg/mL stricturing Crohn’s disease patients’ serum exosomes (CDSE).** Two hours later, elafin was added and further incubated for 24 hours. n = 4 patients; 4 independent experiments. Ordinary one-way ANOVA with Tukey test. (*C*) Fresh human colonic tissues from 4 colon cancer patients were incubated in serum-free RPMI1640 medium with or without 100 μg/mL CDSE. Two hours later, elafin (1 μg/mL) was added and incubated for 24 hours. n = 6 patients. Ordinary one-way ANOVA with Tukey test. (*D–F*) Primary stricturing CD-HIF were pretreated with or without 100 μg/mL CDSE. Two hours later, elafin was added and further incubated for 24 hours. ProCOL1A1, COL1A2, and fibronectin in cell lysates and hydroxyproline and C-telopeptide of type I collagen/ICTP in conditioned media were determined by ELISA. n = 4 patients; 4 independent experiments. Ordinary one-way ANOVA with Tukey test.

CDSE carries CD stricture-related mediators that induce fibrogenesis in intestinal fibroblasts.^6^ CDSE induced pro-collagen I alpha 1 expression with modest COL1A2 protein induction in CD-HIF (Figure 1*A* and *B*). Similarly, CDSE pretreatment induced COL1A1 and COL1A2 mRNA expression in fresh colonic tissues from colon cancer patients (Figure 1*C*). Elafin abolished the induction of collagen expression in CDSE-pretreated CD-HIF and colonic tissues (Figure 1*A–C*).

**Figure 2:**
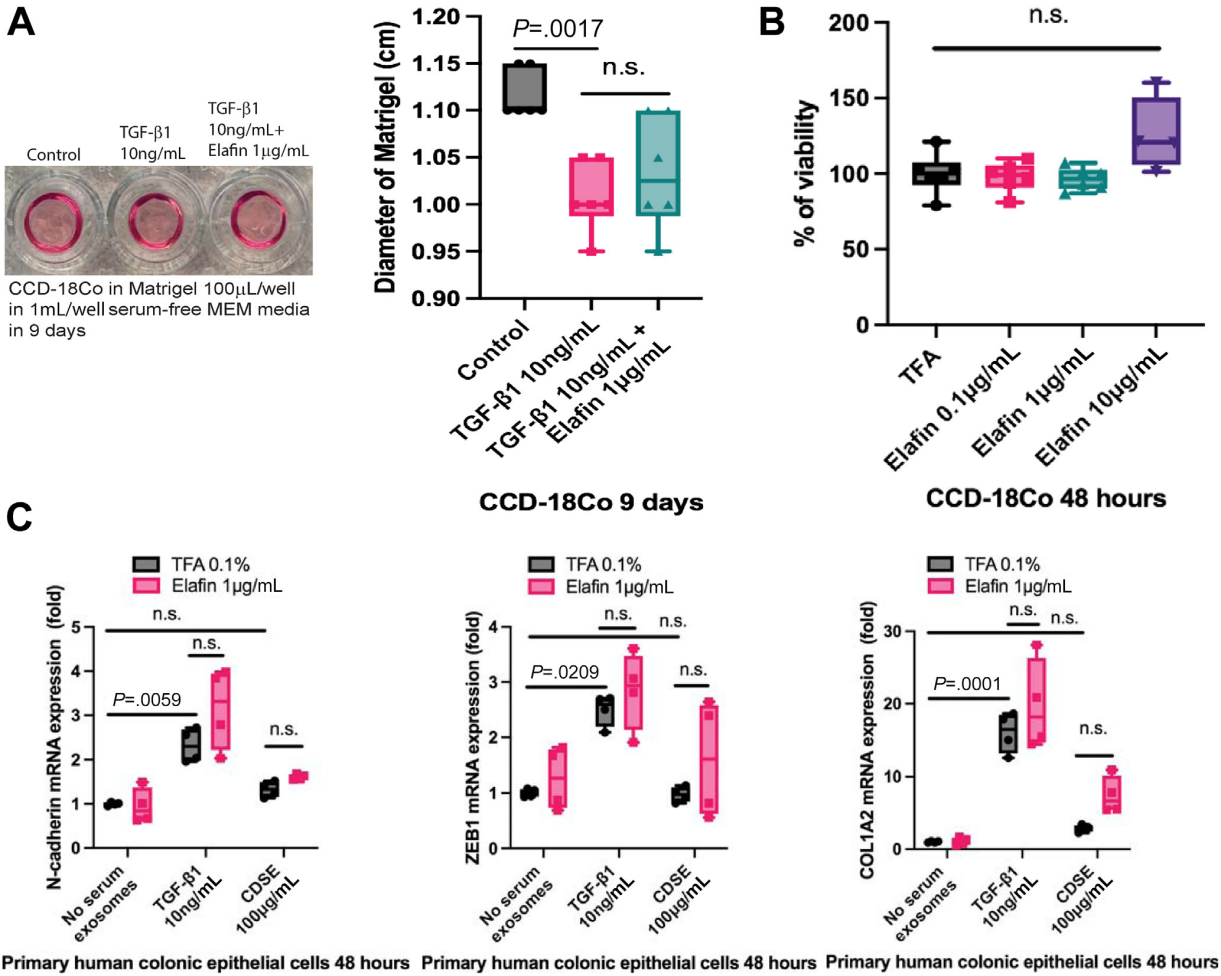
**(*A*) Extracellular matrix (ECM) contraction assay.** Human colonic CCD-18Co fibroblasts were embedded in 100 μL/well of Matrigel (Corning #356234) and covered by 1 mL/well serum-free minimal essential medium (MEM). TGF-β1 (10 ng/mL) and elafin (1 μg/mL) were added to the medium on day 0. The diameter of the Matrigel was measured on day 9. TGF-β1 treatment significantly reduced the Matrigel diameter, indicating increased ECM stiffness. Shrinkage was unaffected by elafin. Results were pooled from 3 independent experiments. Ordinary one-way ANOVA with Tukey test. (*B*) Human colonic CCD-18Co fibroblasts were treated with elafin for 48 hours, followed by addition of MTS assay reagent (G5421; Promega, Madison, WI). Absorbance was determined at 490 nm. Results were pooled from 4 independent experiments. Ordinary one-way ANOVA with Tukey test. (*C*) Serum-starved primary human colonic epithelial cells were pretreated with TGF-β1 (10 ng/mL) or CDSE (100 μg/mL) for 2 hours and then incubated with elafin for 48 hours. N-cadherin, ZEB1, and COL1A2 mRNA expression was determined by real-time reverse transcription polymerase chain reaction. TGF-β1, but not CDSE, induced N-cadherin, ZEB1, and COL1A2 mRNA expression, unaffected by elafin. Results were pooled from 4 independent experiments. Ordinary one-way ANOVA with Tukey test.

Elafin did not affect the expression of extracellular matrix protein fibronectin and secretion of collagen degradation products (hydroxyproline and C-telopeptide of type I collagen/ICTP) in CDSE-treated CD-HIF (Figure 1*D–F*). In addition, elafin did not affect extracellular matrix contraction and cell viability of colonic fibroblasts (Figure 2*A* and *B*). Transforming growth factor beta 1 (TGF-β1), but not CDSE, induced N-cadherin, zinc finger E-box-binding homeobox 1 (ZEB1), and COL1A2 mRNA expression (Figure 2*C*). N-cadherin and ZEB1 are involved in epithelial-mesenchymal transition.^30^^,^^31^ This epithelial-mesenchymal transition–like response was not affected by elafin treatment (Figure 2*C*). These findings suggested that elafin only regulates collagen synthesis in intestinal fibroblasts.

### Activated Intestinal Fibroblasts Secreted Cathepsin S

**Figure 3:**
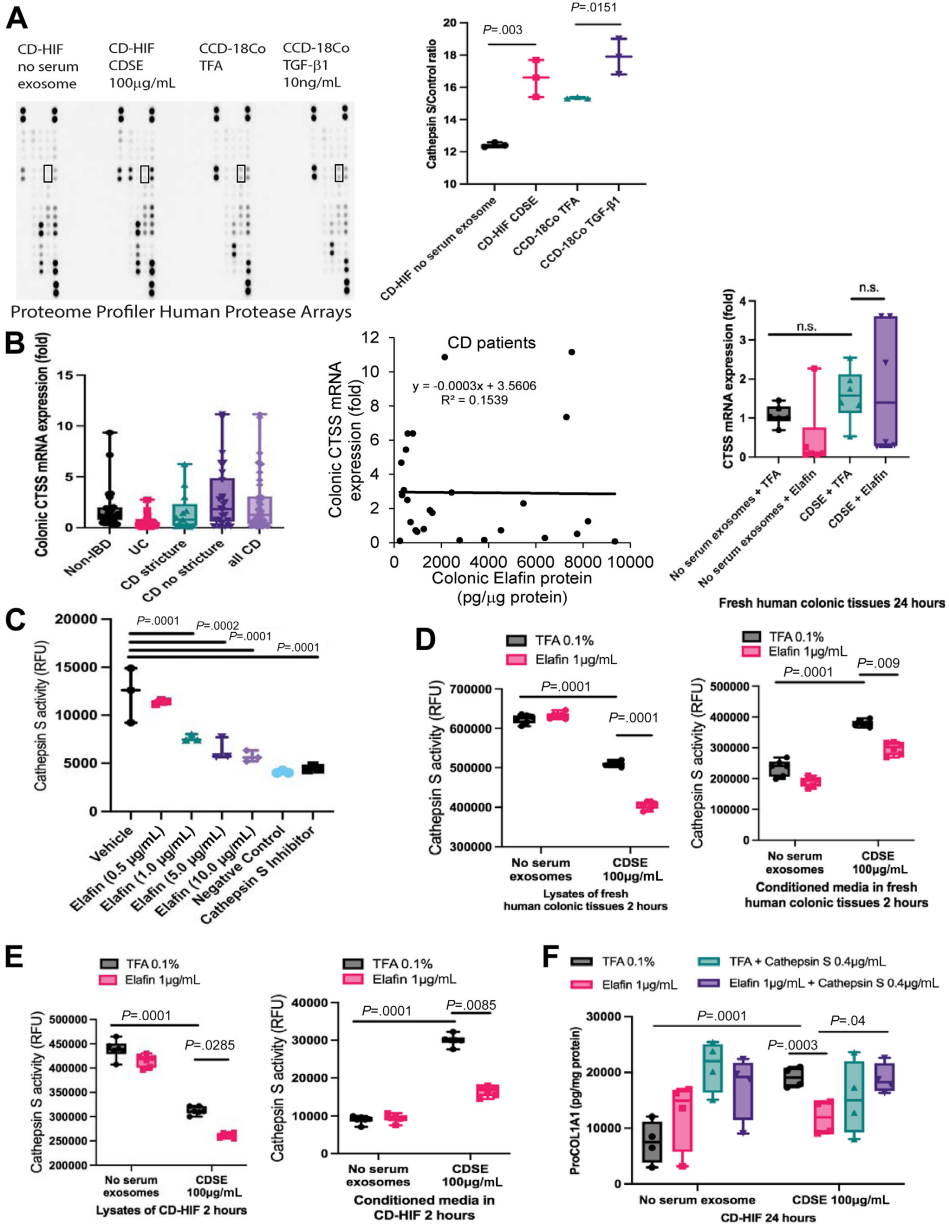
**(*A, left panel*) Serum-starved CD-HIF were treated with 100 μg/mL CDSE for 2 hours.** Serum-starved CCD-18Co colonic fibroblasts were treated with 10 ng/mL TGF-β1 for 2 hours. Conditioned media were loaded to Proteome Profiler Human Protease Arrays (ARY021B; R&D Systems). A Bio-Rad ChemiDoc Imaging system captured the images. The *rectangles* highlighted the cathepsin S expression. (*A, right panel*) Quantification of cathepsin S signals (B7-8) and control signals (A1-2 and E1-2) using Bio-Rad Image Lab Software. Results were pooled from 3 independent experiments. Student *t* test was used to compare no serum exosome and CDSE groups. (*B, left panel*) Colonic cathepsin S mRNA expression in 40 non-IBD, 52 UC, 28 non-stricturing CD, and 15 stricturing CD patients was determined by real-time reverse transcription polymerase chain reaction. Ordinary one-way ANOVA test did not find any significant differences. (*B, middle panel*) Colonic cathepsin S mRNA (CTSS) expression in 43 CD patients is not correlated with colonic elafin mRNA expression. (*B, right panel*) Fresh human colonic tissues from 4 colon cancer patients were incubated in serum-free RPMI1640 media with or without 100 μg/mL CDSE. Two hours later, elafin (1 μg/mL) was added and incubated for 24 hours. Ordinary one-way ANOVA test did not find any significant differences. (*C*) Cathepsin S activity assay was performed by incubating 2 μL of CS substrate (200 μmol/L final concentration), 94 μL CS reaction buffer, 2 μL cathepsin S inhibitor provided by the assay kit, 1 μL cathepsin S (0.4 μg/mL final concentration), and 1 μL elafin (0.5–10 μg/mL final concentration) at 37^o^C for 1 hour. Cathepsin S activity was represented by relative fluorescence units (RFU). Results were pooled from 3 independent experiments. Ordinary one-way ANOVA with Tukey test. (*D*) Fresh human colonic tissues were pretreated with 100 μg/mL CDSE for 2 hours, followed by elafin 1 μg/mL for 2 hours. Conditioned media were collected. Each piece of tissue was homogenized in 500 μL CS cell lysis buffer. Next, 50 μg of tissue lysate supernatants in 50 μL CS lysis buffer or 50 μL of conditioned media were mixed with 2 μL CS substrates (200 μmol/L final concentration) and 48 μL CS reaction buffer and incubated for 1 hour. Cathepsin S activity was represented by relative fluorescence units (RFU). n = 6 patients. Ordinary one-way ANOVA with Tukey test. (*E*) CD-HIF in 96-well plates were pretreated with 100 μg/mL CDSE for 2 hours, followed by elafin 1 μg/mL for 2 hours. Conditioned media were collected. Cells were then lysed in 200 μL/well CS cell lysis buffer. Next, 50 μg of cell lysates in 50 μL CS lysis buffer or 50 μL of conditioned media were mixed with 2 μL of CS substrate (200 μmol/L final concentration) and 48 μL CS reaction buffer and incubated at 37^o^C for 1 hour. Cathepsin S activity was represented by relative fluorescence units (RFU). Results were pooled from 6 independent experiments. Ordinary one-way ANOVA with Tukey test. (*F*) Serum-starved CD-HIF were pretreated with either 0.1% TFA r 0.4 μg/mL cathepsin S (1183-CY-010; R&D Systems) for 30 minutes, followed by 100 μg/mL CDSE. Two hours later, elafin (1 μg/mL) was added and incubated for 24 hours. ProCOL1A1 protein was determined by ELISA. Results were pooled from 4 experiments. Ordinary one-way ANOVA with Tukey test.

We next attempted to discover the upstream target of elafin. Elafin inhibits protease activity, but the fibrosis-mediating protease in intestinal fibroblasts was previously unknown. Protease arrays showed that both CDSE and TGF-β1 induced cathepsin S secretion in the conditioned media of CD-HIF and CCD-18Co fibroblasts, respectively (Figure 3*A*). TGF-β1 activates CCD-18Co fibroblasts with increased collagen synthesis.^32^ Cathepsin S is a cysteine protease that can degrade elastin.^33^

Colonic cathepsin S mRNA (CTSS) expression does not correlate with IBD, intestinal stricture, or colonic elafin protein expression in CD patients (Figure 3*B*, left and middle panels). In addition, elafin treatment did not affect CTSS mRNA expression in CDSE-treated fresh human colonic tissues (Figure 3*B*, right panel).

### Elafin Inhibited Fibrogenesis by Reducing Cathepsin S Activity

We determined the direct interactions between elafin and cathepsin S proteins in cell-free conditions. Interestingly, elafin directly inhibited cathepsin S enzymatic activity in a dose-dependent manner (Figure 3*C*). The inhibitory concentrations against cathepsin S (1–10 μmol/L) were similar to the anti-fibrogenic concentrations of elafin in CDSE-treated CD-HIF (Figure 1*A*). CDSE reduced cathepsin S activity in lysates but increased cathepsin S activity in conditioned media of fresh human colonic tissues and CD-HIF (Figure 3*D* and *E*), suggesting that active cathepsin S was secreted into the conditioned media. Elafin reduced cathepsin S activity in lysates and conditioned media of CDSE-pretreated fresh human colonic tissues and CD-HIF (Figure 3*D* and *E*). Elafin might mediate the anti-fibrogenic effect by inhibiting cathepsin S activity as the addition of cathepsin S reversed elafin-mediated inhibition of ProCOL1A1 expression in CDSE-pretreated CD-HIF (Figure 3*F*).

### Elafin Mediated Anti-Fibrogenic Effect via Protease-Activated Receptor 2 Inhibition

**Figure 4:**
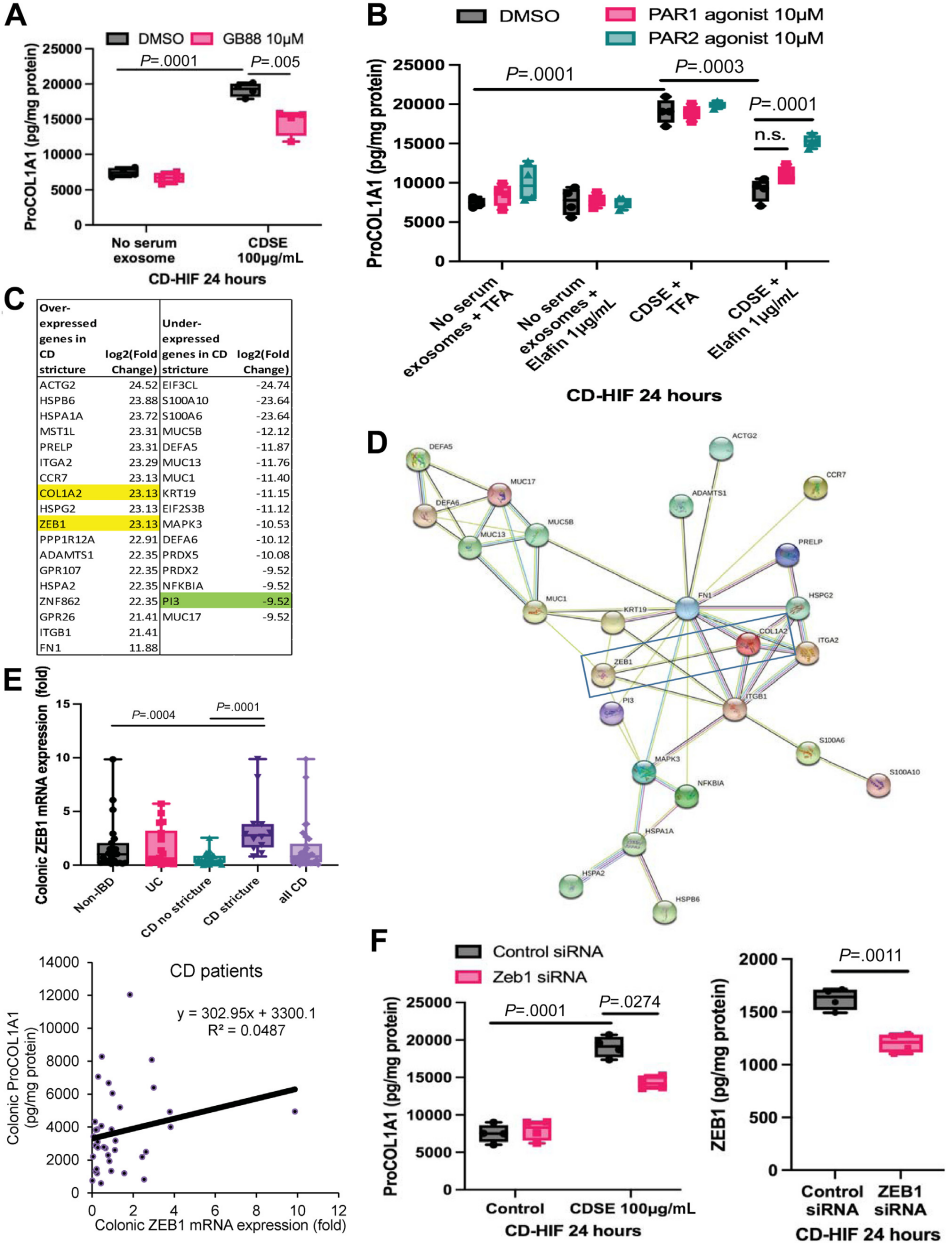
**(*A* and *B*) Serum-starved CD-HIF were pretreated with 0.8% DMSO, 10 μmol/L PAR2 inhibitor GB88 (HY-120261; MCE), 10 μmol/L PAR1 agonist TRAP-6 (HY-P0078; MCE), or 10 μmol/L PAR2 agonist SLIGKV-NH**_**2**_**(HY-P0283; MCE).** An hour later, the fibroblasts were exposed to 100 μg/mL CDSE. Two hours later, elafin (1 μg/mL) was added and incubated for 24 hours. Results were pooled from 4 experiments. Ordinary one-way ANOVA with Tukey test. (*C*) The most differentially expressed genes found in whole-transcriptome RNA sequencing in the colonic tissues of 2 stricturing and 2 non-stricturing CD patients. (*D*) STRING database analysis shows protein interaction association between the most differentially expressed genes in stricturing versus non-stricturing CD patients. (*E, upper panel*) Colonic ZEB1 mRNA expression in 40 non-IBD, 52 UC, 28 non-stricturing CD, and 15 stricturing CD patients. Stricturing CD patients have significantly higher colonic ZEB1 mRNA expression than non-stricturing CD patients. Ordinary one-way ANOVA with Tukey test. (*E, lower panel*) Positive correlation between colonic ZEB1 mRNA and collagen (ProCOL1A1) protein expression in 43 CD patients. (*F*) Serum-starved CD-HIF were transfected with either control (sc-37007; Santa Cruz Biotechnology, Dallas, TX) or ZEB1 (sc-38643; Santa Cruz Biotechnology) siRNA via lipofectamine 3000 overnight, followed by 100 μg/mL CDSE for 24 hours. ZEB1 and ProCOL1A1 proteins were measured by ELISA. Results were pooled from 4 experiments. Ordinary one-way ANOVA with Tukey test for left panel. Student *t* test was used to compare ZEB1 expression between control siRNA and ZEB1 siRNA groups on right panel.

Because cathepsin S activates protease-activated receptor 2 (PAR2) activity,^34^, ^35^, ^36^ we further determined the involvement of PAR2 in the anti-fibrogenic effect of elafin. PAR2 inhibitor GB88 significantly reduced ProCOL1A1 expression in CDSE-pretreated CD-HIF (Figure 4*A*). The elafin-mediated inhibition of ProCOL1A1 expression was abolished by a PAR2 agonist but not a PAR1 agonist (Figure 4*B*). These experiments suggested that elafin inhibits cathepsin S and then PAR2 activity, leading to reduced fibrogenesis.

### Zinc Finger E-Box-Binding Homeobox 1 Is a Target in Intestinal Fibrosis

Whole-transcriptome RNA sequencing showed different colonic gene expressions in stricturing and non-stricturing CD patients.^6^ To discover the downstream target of elafin, we ranked the differentially expressed genes (Figure 4*C*). STRING database analysis showed that ZEB1 is functionally associated with COL1A2 (Figure 4*D*) because ZEB1 regulates collagen promoter activity and expression.^37^^,^^38^ Stricturing CD patients had significantly higher colonic ZEB1 mRNA expression than non-IBD and non-stricturing CD patients (Figure 4*E*, upper panel). In addition, colonic ZEB1 mRNA expression is positively correlated with ProCOL1A1 protein expression in CD patients (Figure 4*E*, lower panel), suggesting its association with intestinal fibrosis. ZEB1 regulates fibrogenesis as siRNA-mediated ZEB1 inhibition reduced collagen expression in CDSE-treated CD-HIF (Figure 4*F*, left panel). Transfection of ZEB1 siRNA was efficient in reducing ZEB1 protein expression (Figure 4*F*, right panel).

### Elafin Suppressed Collagen Synthesis via Zinc Finger E-Box-Binding Homeobox 1 Inhibition

**Figure 5:**
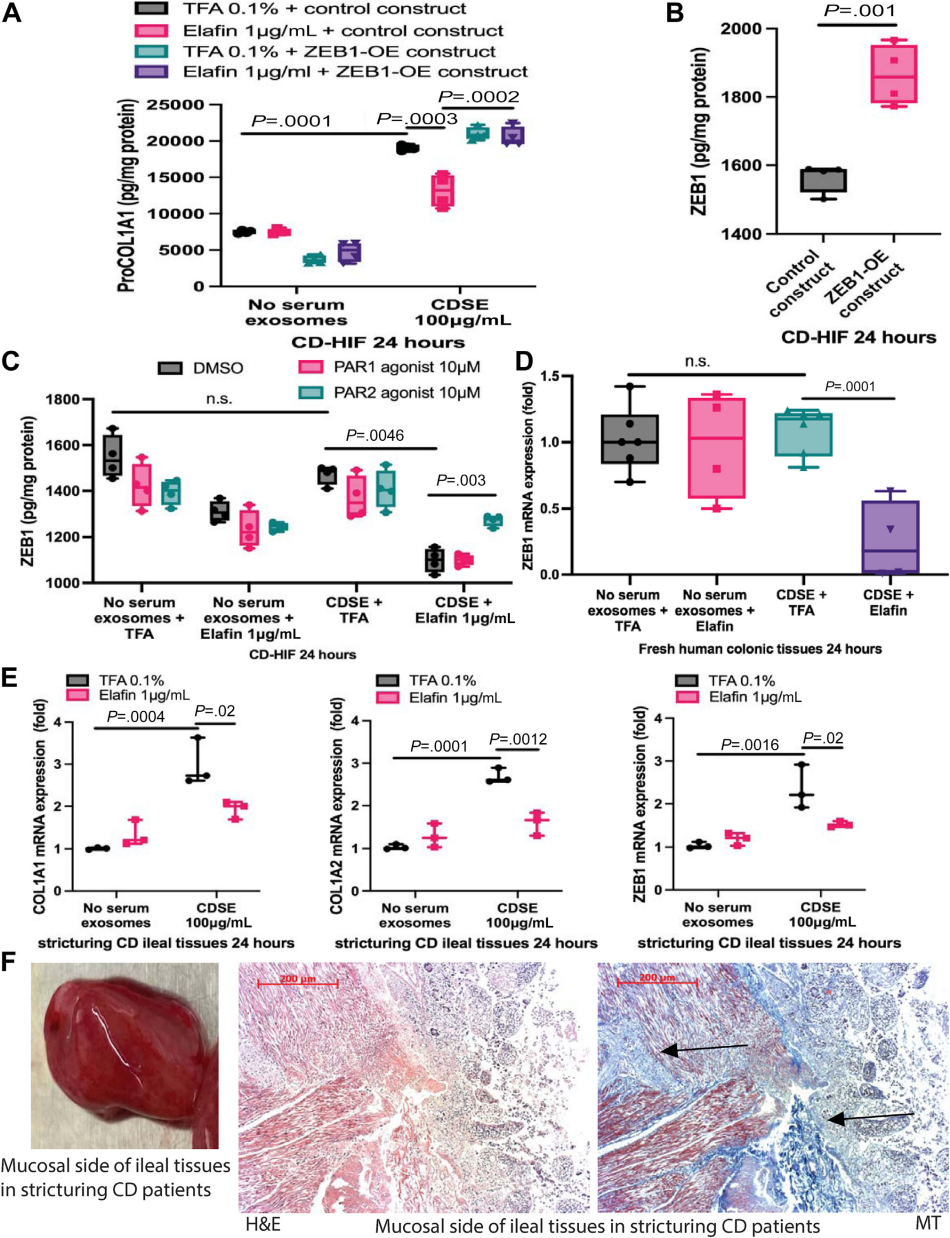
**(*A*) Serum-starved CD-HIF were transfected with either control or ZEB1-overexpressing construct via lipofectamine 3000 overnight.** Fibroblasts were then pretreated with 100 μg/mL CDSE. Two hours later, elafin (1 μg/mL) was added and incubated for 24 hours. Results were pooled from 4 independent experiments. Ordinary one-way ANOVA with Tukey test. (*B*) Efficiency of ZEB1-overexpressing construct transfection was determined by ELISA. Results were pooled from 4 experiments. Student *t* test was used to compare ZEB1 expression between control construct and ZEB1-overexpressing construct groups. (*C*) Serum-starved CD-HIF were pretreated with DMSO, 0.4 μg/mL cathepsin S, 10 μmol/L PAR1 agonist TRAP-6 (HY-P0078; MCE), or 10 μmol/L PAR2 agonist (HY-P0283; MCE) for 60 minutes, followed by addition of 100 μg/mL CDSE. Two hours later, some groups were treated with elafin (1 μg/mL) and incubated for 24 hours. Results were pooled from 4 independent experiments. Ordinary one-way ANOVA with Tukey test. (*D*) Fresh human colonic tissues from 4 colon cancer patients were incubated in serum-free RPMI1640 media with or without 100 μg/mL CDSE. Two hours later, elafin was added and further incubated for 24 hours. Results were pooled from 4 independent experiments. Ordinary one-way ANOVA with Tukey test. (*E*) Fresh human ileal tissues from 3 stricturing CD patients were incubated in serum-free RPMI1640 media with or without 100 μg/mL CDSE. Two hours later, elafin (1 μg/mL) was added and incubated for 24 hours. COL1A1, COL1A2, and ZEB1 mRNA expression were determined by real-time reverse transcription polymerase chain reaction. Ordinary one-way ANOVA with Tukey test. (*F*) Macroscopic and microscopic morphology of the fresh stricturing ileal tissue. Intense collagen deposition is found in the mucosal layer.

Although ZEB1 overexpression did not augment collagen synthesis in CDSE-pretreated CD-HIF, it abolished the anti-fibrogenic effect of elafin (Figure 5*A*). Transfection of the ZEB1-overexpressing construct efficiently increased ZEB1 protein expression (Figure 5*B*). Elafin might reduce ZEB1 protein expression via PAR2 inhibition because this inhibition was reversed by a PAR2 agonist (Figure 5*C*). These experiments indicated that elafin inhibits collagen synthesis via sequential inhibition of cathepsin S and PAR2 activity, followed by ZEB1 expression in intestinal fibroblasts.

Like CD-HIF, elafin inhibited ZEB1 mRNA expression in CDSE-treated fresh human colonic tissues (Figure 5*D*). However, intestinal strictures can occur in the ileum. Elafin inhibited CDSE-induced collagen and ZEB1 mRNA expression in fresh ileal tissues from stricturing CD patients (Figure 5*E* and *F*), suggesting that elafin may be useful for resolving ileal strictures in CD patients. Therefore, it is justified to use 3 mouse models of intestinal fibrosis to validate the mechanistic relationships between elafin, cathepsin S, PAR2, and Zeb1 in vivo.

### Elafin Overexpression Inhibited Intestinal fibrosis in SAMP1/YitFc, Salmonella, and Trinitrobenzene Sulfonic Acid Mouse Models

**Figure 6:**
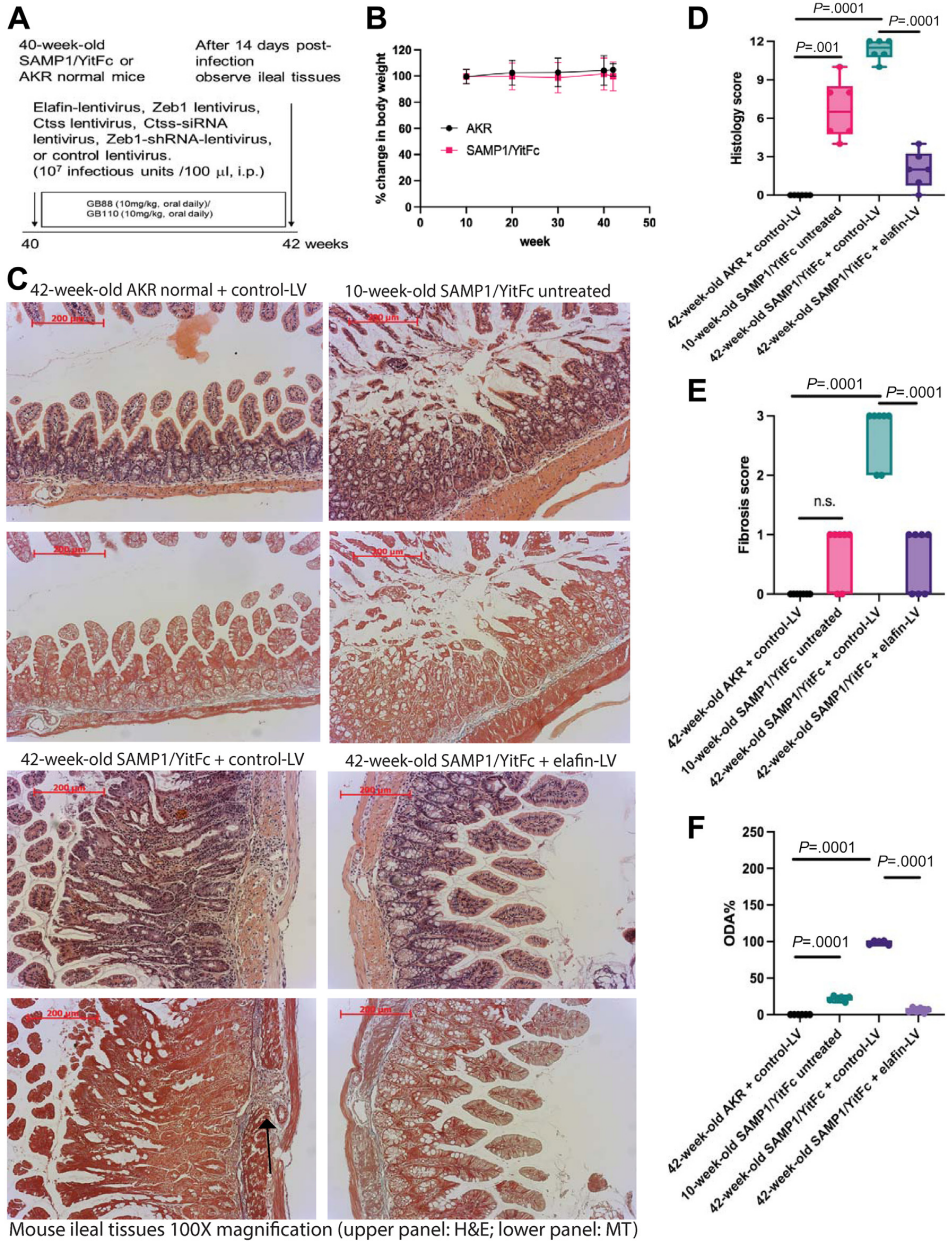
**(*A*) Experimental plan.** Control lentivirus, elafin-overexpressing lentiviruses, Ctss-overexpressing lentivirus, Ctss-siRNA lentivirus, Zeb1-shRNA lentivirus, and Zeb1-overexpressing lentivirus were injected into SAMP1/YitFc mice intraperitoneally once at 40 weeks of age. In addition, PAR2 agonist GB110 or PAR2 inhibitor GB88 was given via oral gavage from 40 to 42 weeks of age. Non-fibrotic 10-week-old SAMP1/YitFc mice and parental control 42-week-old AKR strain mice were used for comparison. Ileal tissues were collected for analysis 2 weeks after lentiviral injection. (*B*) Body weight. Six mice per group. Mean ± standard deviation. (*C*) H&E staining (*upper panels*) and Masson Trichrome (MT) staining (*lower panels*) of ileal tissues from 10 to 42 weeks of age. Blue color in MT staining (*arrows*) indicated collagen deposition in lamina propria. (*D*) Ileal histology scores. (*E*) Ileal fibrosis scores. (*F*) Ileal overall disease activities. Six mice per group. Ordinary one-way ANOVA with Tukey tests.

SAMP1/YitFc mice are an established mouse model for studying CD because they develop spontaneous CD-like ileitis with preexisting ileal fibrosis at 40 weeks of age (Figure 6*A*).^28^ There was no significant change in body weight in SAMP1/YitFc mice from 10 to 42 weeks of age (Figure 6*B*). In addition, the young 10-week-old SAMP1/YitFc mice developed spontaneous ileitis but not fibrosis (Figure 6*C*).

There is no consensus approach to characterize intestinal fibrosis. Therefore, we attempted to include commonly reported fibrosis- and inflammation-related parameters to compare disease activity between groups and models. Lentiviral elafin overexpression reversed preexisting ileal fibrosis with lowered histology score, fibrosis score, and overall disease activity (ODA) in SAMP1/YitFc mice from 40 to 42 weeks (Figure 6*C–F*). The disease activity parameters of SAMP1/YitFc mice are shown in Table 1.

**Table 1:** Ileal Overall Disease Activity and Gene Expression Profile in SAMP1/YitFc Model

Ileal	HS	FS	Col1a2	Col3a1	Zeb1	Vim	Acta2	Tnf	Emr1	ODA%	Ctss
	score	score	mRNA	mRNA	mRNA	mRNA	mRNA	mRNA	mRNA	Mean ± SD	mRNA
42-week-old AKR + control-LV											
Mean	0.0	0.0	1.1	1.1	1.1	1.1	0.8	1.1	1.1		1.0
SD	0.0	0.0	0.5	0.5	0.5	0.5	0.1	0.1	0.5		0.2
%	0	0	0	0	0	0	0	0	0	**0**	
10-week-old SAMP1Yit/Fc Untreated											
Mean	6.7	0.7	2.3	5.6	5.6	112.9	0.5	0.6	2.5		0.9
SD	2.3	0.5	4.5	6.3	6.3	117.3	0.1	0.2	1.4		0.4
%	59	26	6	20	21	23	0	0	40	**22 ± 3.3**	
42-week-old SAMP1Yit/Fc+ Control-LV											
Mean	11.3	2.7	21.0	24.0	23.0	486.0	2.5	2.6	4.5		1.0
SD	0.8	0.5	6.3	11.2	5.5	85.0	0.1	0.1	2.2		0.1
%	100	100	100	100	100	100	100	100	100	**100 ± 2.7**	100
compared with AKR											
*P* value	.0001	.0001	.0001	.0001	.0001	.0001	.0413	.0164	.0001		NS
42-week-old SAMP1Yit/Fc+ elafin-LV											
Mean	2.0	0.6	0.7	1.6	2.4	40.6	0.6	0.4	0.2		0.4
SD	1.4	0.5	0.3	0.3	0.6	18.0	0.1	0.1	0.1	**6 ± 3.1**	0.2
%	18	21	0	2	6	8	0	0	0		46
compared with SAMP control-LV											
*P* value	.0001	.0001	.0001	.0002	.0001	.0001	.0138	.0003	.0001	**.0001**	.002
42-week-old SAMP1Yit/Fc+ elafin-LV + Ctss-OE-LV											
Mean	10.8	2.6	6.3	23.0	164.3	404.0	8.5	3.6	5.9		0.8
SD	0.8	0.5	2.5	15.6	11.5	93.0	1.7	0.2	0.03		0.2
%	96	95	26	96	745	83	453	165	141	**211 ± 2.1**	80
compared with SAMP elafin-LV											
*P* value	.0001	.0001	.045	.0004	.0001	.0001	.0001	.0001	.0001	**.0001**	.040
42-week-old SAMP1Yit/Fc+ elafin-LV + GB110											
Mean	10.3	2.4	2.3	100.3	98.3	254.6	5.6	1.3	4.6		N/A
SD	0.8	0.5	0.3	11.1	9.1	46.2	1.5	0.4	0.1	**169 ± 2.3**	
%	91	89	6	433	444	52	284	15	104		
compared with SAMP elafin-LV											
*P* value	.0001	.0001	NS	.0001	.0001	.0001	.0001	NS	.0001	**.0001**	
42-week-old SAMP1Yit/Fc+ elafin-LV + Zeb1-OE-LV											
Mean	9.5	2.6	6.4	0.9	96.0	185.5	2.7	2.9	4.6		N/A
SD	1.6	0.5	0.3	0.2	3.4	11.5	0.9	1.2	1.2		
%	84	95	27	0	434	38	110	118	102	**112 ± 3.2**	
compared with SAMP elafin-LV											
*P* value	.0001	.0001	.03	NS	.0001	.0020	.0042	.0017	.0001	**.0001**	
42-week-old SAMP1Yit/Fc+ Ctss-siRNA-LV											
Mean	4.8	1.4	5.4	14.1	1.2	12.1	3.4	2.3	5.0		0.5
SD	0.8	0.5	1.7	4.3	0.7	11.3	0.3	1.6	1.3		0.2
%	43	53	22	57	1	2	152	81	114	**58 ± 2.3**	48
compared with SAMP control-LV											
*P* value	.0001	.0027	.0001	NS	.0001	.0001	NS	NS	NS	**.0001**	.01
42-week-old SAMP1Yit/Fc+ GB88											
Mean	3.5	1.1	6.5	6.5	10.9	6.4	2.7	1.1	3.7		N/A
SD	0.5	0.7	4.2	3.3	2.6	1.8	0.7	0.6	0.5		
%	31	42	27	24	45	1	112	0	77	**39 ± 2.9**	
compared with SAMP control-LV											
*P* value	.0001	.0001	.0001	.0072	.0149	.0001	NS	.0189	NS	**.0001**	
42-week-old SAMP1Yit/Fc+ Zeb1-shRNA-LV											
Mean	4.5	1.1	12.8	11.3	5.1	12.0	2.5	1.8	5.2		N/A
SD	1.0	0.7	4.2	3.3	2.6	1.8	1.0	0.1	0.7		
%	40	42	59	45	18	2	100	48	119	**51 ± 3.8**	
compared with SAMP control-LV											
*P* value	.0001	.0001	.0059	NS	.0001	.0001	NS	NS	NS	**.0001**	

SD, standard deviation.

**Figure 7:**
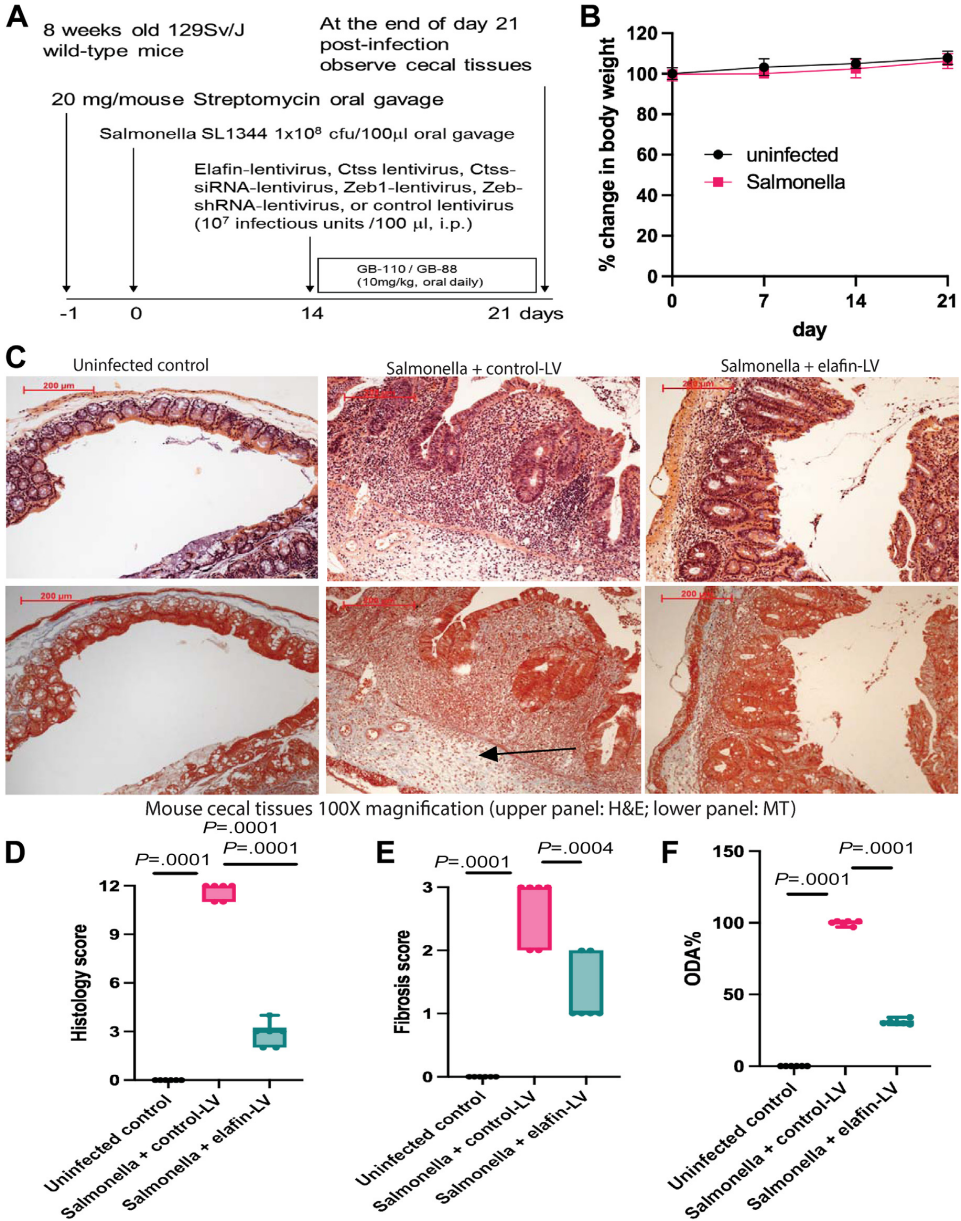
**(*A*) Experimental plan.** Eight-week-old male and female 129Sv/J mice were administered 20 mg streptomycin via oral gavage. Twenty-four hours later, mice were orally infected with *Salmonella typhimurium* SL1344 strain (1 × 10^8^ colony-forming units) to induce cecal fibrosis. In addition, some mice received single intraperitoneal injection (10^7^ infectious units/mouse) of control lentivirus or elafin-overexpressing lentiviruses on day 14. Cecal tissues were collected for analysis on day 21. (*B*) No significant change in body weight was noticed throughout the disease course in the infected mice. Six mice per group. Mean ± standard deviation. (*C*) H&E staining (*upper panels*) and Masson Trichrome (MT) staining (*lower panels*) of cecal tissues on day 21. Blue color in MT staining (*arrows*) indicated collagen deposition in cecal lamina propria of *Salmonella*-infected mice. (*D*) Cecal histology scores. (*E*) Cecal fibrosis scores. (*F*) Cecal overall disease activities. Lentiviral elafin expression reversed cecal fibrosis in *Salmonella*-infected mice. Six mice per group. Ordinary one-way ANOVA with Tukey tests.

Similarly, *Salmonella* infection induced cecitis, followed by cecal fibrosis with mucosal disruption, immune cell infiltration, and collagen deposition on day 21 (Figure 7*A*).^39^ There was no significant change in body weight in the infected mice from day 0 to day 21 (Figure 7*B*). Notably, lentiviral elafin overexpression (from day 14 to 21) ameliorated cecal fibrosis with lowered histology score, fibrosis score, and ODA in the infected mice (Figure 7*C–F*). The disease activity parameters of *Salmonella*–infected mice are shown in Table 2.

**Table 2:** Cecal Overall Disease Activity and Gene Expression Profile in Salmonella Model

Cecal	HS	FS	Col1a2	Zeb1	Vim	Acta2	Tnf	Emr1	ODA%	Ctss
	score	score	mRNA	mRNA	mRNA	mRNA	mRNA	mRNA	Mean ± SD	mRNA
Uninfected control										
Mean	0.0	0.0	1.0	1.0	1.1	1.0	1.0	1.0	0	1.1
SD	0.0	0.0	0.2	0.3	0.4	0.2	0.3	0.1		0.3
%	0	0	0	0	0	0	0	0		
Salmonella + control-LV										
Mean	11.7	2.8	5.3	3.8	7.9	2.2	216.6	22.5	100 ± 1.5	3.9
SD	0.5	0.4	0.3	0.4	2.9	0.2	40.0	2.3		0.7
%	100	100	100	100	100	100	100	100		100
Compared with uninfected										
*P* value	.0001	.0001	.0001	.0041	.0294	.0003	.0001	.0002		.0001
Salmonella + elafin-LV										
Mean	2.8	1.2	1.2	1.2	3.1	0.7	100.0	8.8	23 ± 1.6	1.8
SD	0.8	0.4	1.0	0.5	2.0	0.2	9.0	1.5		0.4
%	24	41	5	4	30	0	46	36		24
Compared with SAL + control-LV										
*P* value	.0001	.0004	.0002	.0084	NS	.0001	.0085	.0490	.0001	.0003
Salmonella + Ctss-OE-LV + elafin-LV										
Mean	10.5	2.8	11.6	3.8	7.4	1.6	87.6	12.6	97 ± 2.3	3.4
SD	0.8	0.4	3.2	1.1	1.6	0.6	75.4	4.6		1.2
%	90	100	245	101	94	49	40	54		82
Compared with SAL + elafin-LV										
*P* value	.0001	.0001	.0001	.008	NS	.0132	NS	NS	.0001	.01
Salmonella + GB110 + elafin-LV										
Mean	10.3	2.5	6.1	7.2	12.7	1.7	139.4	65.5	139 ± 2.4	N/A
SD	1.2	0.5	1.9	1.3	4.9	0.4	58.8	3.2		
%	89	88	117	223	171	61	64	300		
Compared with SAL + elafin-LV										
*P* value	.0001	.0028	.0001	.0001	.0003	.0039	NS	.0001	.0001	
Salmonella + Zeb1-LV + elafin-LV										
Mean	10.3	2.7	8.2	9.7	12.0	1.3	121.2	46.7	140 ± 2.9	N/A
SD	1.4	0.5	1.4	2.8	6.6	0.7	93.0	20.5		
%	89	94	167	314	161	24	56	212		
Compared with SAL + elafin-LV										
*P* value	.0001	.0004	.0001	.0001	.0010	NS	NS	.0001	.0001	
Salmonella + Ctss-siRNA-LV										
Mean	6.8	1.3	1.1	1.4	5.1	0.6	78.7	4.5	30 ± 2.6	0.7
SD	1.2	0.5	0.4	0.2	4.1	0.4	67.8	4.0		0.6
%	59	47	2	14	59	0	36	16		0
Compared with SAL + control-LV										
*P* value	.0001	.0004	.0001	.0196	NS	.0001	.001	.0029	.0001	.0001
Salmonella + GB88 + control-LV										
Mean	4.3	0.8	0.5	1.4	0.9	0.5	3.1	1.9	11 ± 2.7	N/A
SD	1.0	0.4	0.5	0.8	0.4	0.5	2.7	0.3		
%	37	29	0	14	0	0	1	4		
Compared with SAL + control-LV										
*P* value	.0001	.0001	.0001	.019	.0181	.0001	.0001	.0004	.0001	
Salmonella + Zeb1-shRNA-LV + control-LV										
Mean	4.2	0.5	0.7	1.6	1.0	0.5	2.8	0.7	9 ± 2.7	N/A
SD	0.4	0.5	0.3	0.1	0.1	0.2	2.4	0.5		
%	36	18	0	19	0	0	1	0		
Compared with SAL + control-LV										
*P* value	.0001	.0001	.0001	.0434	.0209	.0001	.0001	.0002	.0001	
Salmonella + ABX + control-LV										
Mean	10.00	2.50	4.67	2.45	6.07	1.98	191.52	25.37	86 ± 1.68	N/A
SEM	0.50	0.29	0.19	0.28	0.68	0.16	17.04	3.33		
%	64	88	85	51	74	82	88	113		
Compared with SAL + control-LV										
*P* value	NS	NS	NS	NS	NS	NS	NS	NS		
Salmonella + ABX + elafin-LV										
Mean	3.00	0.75	2.64	2.17	4.81	1.48	97.60	14.38	51 ± 1.62	N/A
SEM	0.71	0.25	0.43	0.31	0.83	0.17	3.09	2.19		
%	26	26	38	41	55	40	45	62		
Compared with SAL + elafin-LV										
*P* value	NS	NS	NS	NS	NS	NS	NS	NS		
Salmonella + miR205-5p-OE-LV + control-LV										
Mean	5.67	0.83	2.80	2.78	5.48	1.69	26.14	8.74	47 ± 0.92	N/A
SEM	0.21	0.17	0.31	0.26	0.34	0.15	3.96	0.94		
%	49	29	41	63	65	57	12	36		
Compared with SAL + control-LV										
*P* value	.0001	.0001	.0001	NS	NS	NS	.0001	.0001	.0001	
Salmonella + miR205-5p-OFF-LV + elafin-LV										
Mean	9.67	2.17	2.79	3.32	5.98	1.56	38.94	10.07	60 ± 0.86	N/A
SEM	0.21	0.17	0.22	0.61	0.91	0.24	5.51	1.24		
%	83	77	41	83	72	46	18	42		
Compared with SAL + elafin-LV										
*P* value	.0001	.0005	.0494	.0439	NS	.0244	.0015	.0205	.0001	

**Figure 8:**
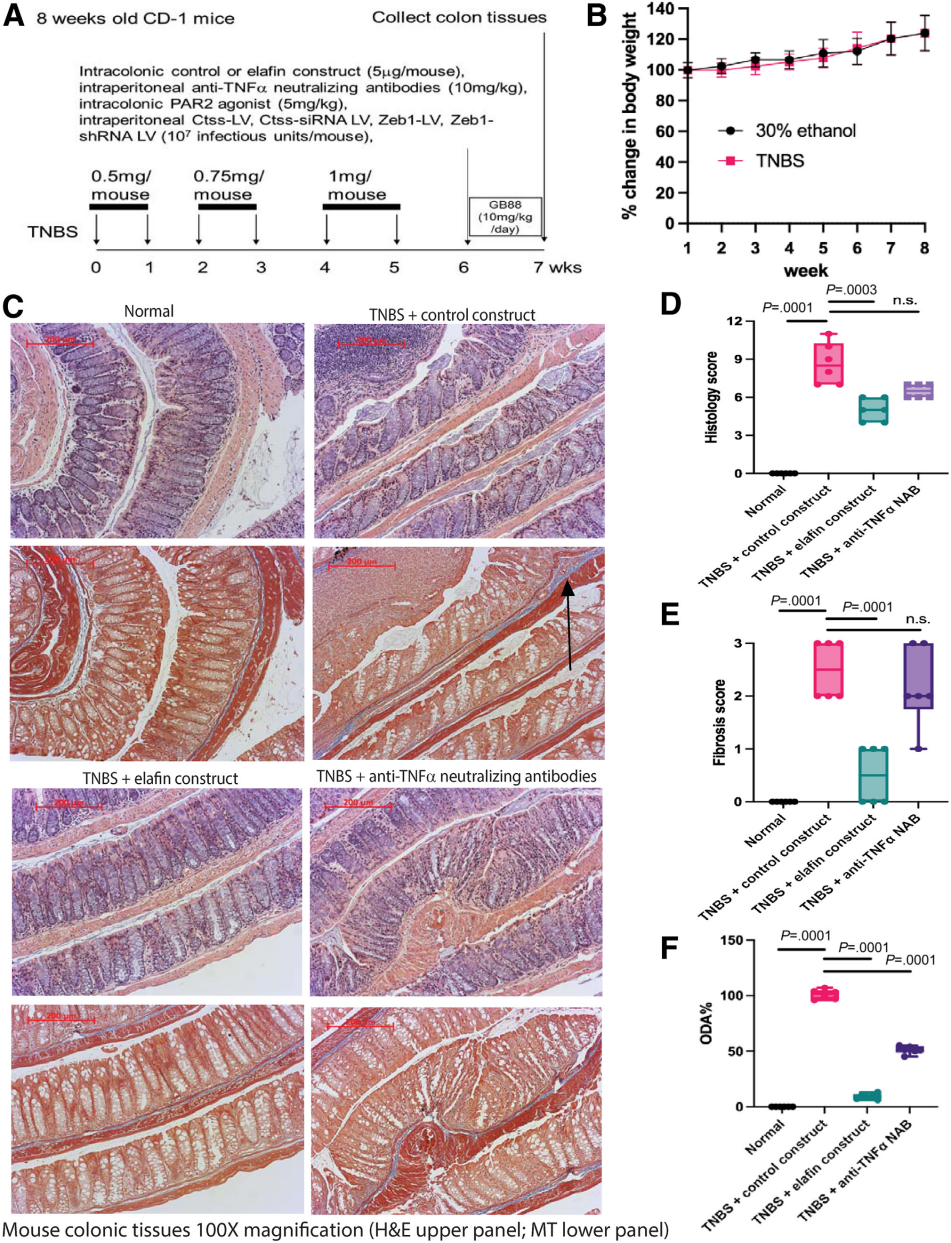
**(*A*) Experimental plan.** Eight-week-old male and female CD-1 mice were injected with 50 μL TNBS solution (to induce colitis) or 30% ethanol (vehicle) via weekly enema 6 times. After last TNBS injection, mice were held for 2 additional weeks to develop colonic fibrosis. Some mice were injected with either control construct or elafin-overexpressing construct intracolonically on day 9 after last TNBS injection. Anti-TNFα neutralizing antibodies were injected intraperitoneally on day 9 after last TNBS injection. (*B*) TNBS induced colitis slowly. No significant change in weight loss was noticed in TNBS-treated mice. Six mice per group. Mean ± standard deviation. (*C*) H&E staining (*upper panels*) and MT staining (*lower panels*) of colonic tissues. Blue color in MT staining (*arrows*) indicated collagen deposition. (*D*) Colonic histology scores. (*E*) Colonic fibrosis scores. (*F*) Colonic overall disease activities. Six mice per group. Ordinary one-way ANOVA with Tukey test.

Multiple intracolonic TNBS injections induced colonic fibrosis (Figure 8*A*)^10^^,^^40^ but did not cause weight loss in mice (Figure 8*B*). Intracolonic transfection of elafin-overexpressing constructs ameliorated colonic fibrosis with lowered histology score, fibrosis score, and ODA in the TNBS-treated mice within 7 days (Figure 8*C–F*). The disease activity parameters of TNBS-treated mice are shown in Table 3. In general, elafin overexpression consistently reduced intestinal fibrosis in these 3 mouse models.

**Table 3:** Colonic Overall Disease Activity and Gene Expression Profile in TNBS Model

Colonic	HS	FS	Col1a2	Col3a1	Zeb1	Vim	Acta2	Tnf	Emr1	ODA%	Ctss
	score	score	mRNA	mRNA	mRNA	mRNA	mRNA	mRNA	mRNA	Mean ± SD	mRNA
Normal											
Mean	0.0	0.0	0.9	0.9	1.0	1.0	1.1	1.1	1.1	0	1.1
SD	0.0	0.0	0.2	0.4	0.3	0.3	0.2	0.3	0.6		0.3
%	0.0	0.0	0.0	0.0	0.0	0.0	0.0	0.0	0.0		
TNBS + control construct											
Mean	8.7	2.5	2.2	2.5	1.7	2.5	2.4	2.0	2.4	100 ± 3.2	0.9
SD	1.6	0.5	0.2	0.3	0.3	0.2	1.0	0.2	1.0		0.2
%	100	100	100	100	100	100	100	100	100		100
Compared with normal											
*P* value	.0001	.0001	.0372	.0059	.0477	.0349	.0019	.0364	.0364	.0001	NS
TNBS + elafin construct											
Mean	5.0	0.5	0.4	0.5	0.6	1.0	0.8	1.1	0.6	9 ± 2.7	0.5
SD	0.9	0.5	0.1	0.2	0.2	0.2	0.1	0.3	0.1		0.2
%	58	20	0	0	0	0	0	5	0		53
Compared with TNBS + control construct											
*P* value	.0003	.0001	.0008	.0002	.0002	.0394	.0001	NS	NS	.0001	.0191
TNBS + anti-TNFα NAB											
Mean	6.5	2.2	1.7	2.5	1.5	1.7	1.4	1.1	0.6	51 ± 3.6	N/A
SD	0.5	0.8	0.2	0.2	0.4	0.4	0.4	0.5	0.4		
%	75	87	66	100	70	43	19	0	0		
Compared with TNBS + control construct											
*P* value	NS	NS	NS	NS	NS	NS	.0249	.0364	.0364	.0001	
TNBS + elafin construct + Ctss-OE-LV											
Mean	11.2	2.7	1.8	2.0	2.2	1.3	1.8	1.4	2.6	85 ± 2.2	1.0
SD	0.8	0.5	0.9	1.1	0.6	0.2	0.3	0.3	0.5		0.2
%	129	107	69	67	170	20	53	32	117		108
Compared with TNBS + elafin construct											
*P* value	.0001	.0001	.0026	.0135	.0001	NS	.0396	NS	NS	.0001	.005
TNBS + elafin construct + PAR2 agonist											
Mean	8.8	2.3	3.3	2.7	2.0	4.0	1.9	0.5	2.9	114 ± 3.6	N/A
SD	1.2	0.5	1.2	1.3	0.4	0.5	0.6	0.2	1.9		
%	102	93	186	113	140	196	61	0	136		
Compared with TNBS + elafin construct											
*P* value	.0002	.0001	.0001	.0001	.0001	.0001	.0154	NS	NS	.0001	
TNBS + elafin construct + Zeb1-LV											
Mean	8.5	2.2	1.7	1.9	1.6	2.6	2.3	0.8	0.8	66 ± 4.0	N/A
SD	1.0	0.8	0.5	1.0	0.3	1.8	0.7	0.2	0.5		
%	98	87	61	64	85	103	93	0	0		
Compared with TNBS + elafin construct											
*P* value	.0007	.0010	.0457	.0194	.0009	.0309	.0002	NS	NS	.0001	
TNBS + Ctss-siRNA-LV											
Mean	6.0	1.2	1.9	1.9	1.3	3.1	1.4	0.7	1.8	57 ± 2.6	0.5
SD	1.4	0.4	1.2	0.7	0.4	1.1	0.3	0.1	0.3		0.1
%	69	47	77	60	39	141	25	0	54		50
Compared with TNBS + control construct											
*P* value	.02	.0038	NS	NS	NS	NS	.0473	.0003	.0003	.0001	.011
TNBS + GB88											
Mean	3.0	0.3	0.7	0.9	1.0	1.7	0.9	1.4	2.6	27 ± 4.2	N/A
SD	1.9	0.5	0.1	0.0	0.5	0.9	0.6	1.0	1.3		
%	35	13	0	0	4	46	0	35	113		
Compared with TNBS + control construct											
*P* value	.0001	.0001	.0091	.0047	NS	NS	.0003	NS	NS	.0001	
TNBS + Zeb1-shRNA-LV											
Mean	3.5	0.5	1.2	1.1	1.0	1.3	0.6	0.9	1.9	20 ± 4.5	N/A
SD	2.0	0.5	0.1	0.0	0.1	0.2	0.2	0.2	0.2		
%	40	20	26	12	0	16	0	0	64		
Compared with TNBS + control construct											
*P* value	.0001	.0001	NS	.0239	.0332	NS	.0001	.0013	.0013	.0001	

Colonic	HS	FS	Col1a2	Col3a1	Zeb1	Vim	Acta2	Tnf	Emr1	ODA%	Ctss
	score	score	mRNA	mRNA	mRNA	mRNA	mRNA	mRNA	mRNA	mean±sd	mRNA
Normal											
Mean	0.0	0.0	0.9	0.9	1.0	1.0	1.1	1.1	1.1	0	1.1
SD	0.0	0.0	0.2	0.4	0.3	0.3	0.2	0.3	0.6		0.3
%	0.0	0.0	0.0	0.0	0.0	0.0	0.0	0.0	0.0		
TNBS + control Eudragit-HPMC											
Mean	8.5	2.3	1.7	2.5	1.5	1.8	2.0	1.7	2.2	99 ± 3.3	1.0
SD	2.1	0.6	0.4	0.5	0.2	0.3	0.4	0.5	0.9		0.2
%	100	100	100	100	100	100	100	100	100		100
TNBS + Elafin Eudragit-HPMC											
Mean	2.8	0.4	0.8	0.5	0.8	1.1	1.2	0.7	1.1	7 ± 2.6	0.4
SD	2.0	0.6	0.1	0.1	0.4	0.5	0.4	0.2	0.6		0.1
%	33	18	0	0	0	0	13	0	0		42
Compared with TNBS + control Eudragit											
*P* value	.0013	.0007	.0001	.0001	.0061	.0234	NS	.0002	.0282	.0001	.0016

Anti-TNFα neutralizing antibodies are widely used for treating intestinal inflammation among IBD patients. For comparison, injection of anti-TNFα neutralizing antibodies partially ameliorated colitis with moderately lowered histology score and ODA (Figure 8*C*, *D*, and *F*). However, this treatment failed to reverse colonic fibrosis because the fibrosis score remained high (Figure 8*C* and *E*).

### Inhibition of Ctss, Protease-Activating Receptor 2, and Zinc Finger E-Box-Binding Homeobox 1 Ameliorated Intestinal Fibrosis in Vivo

**Figure 9:**
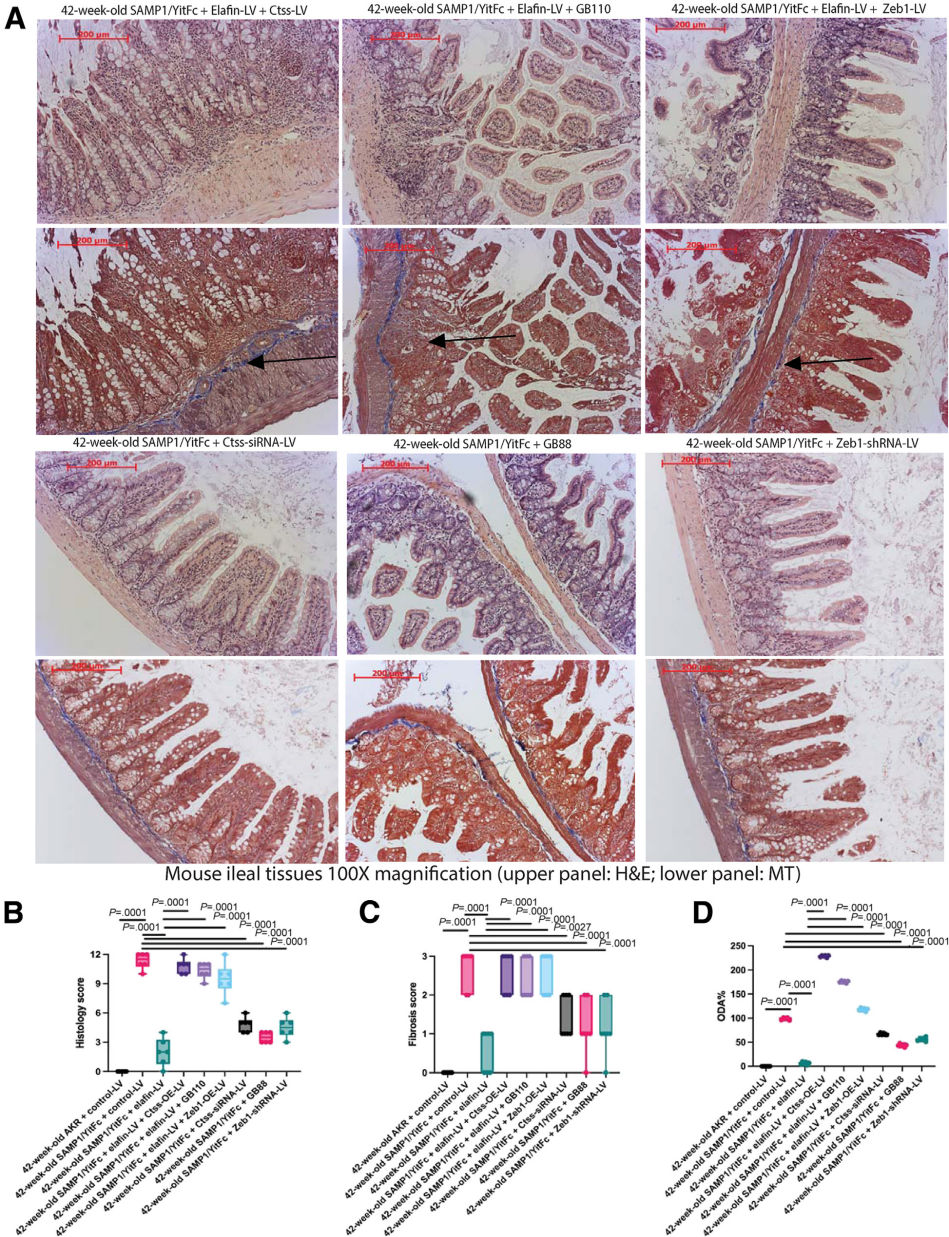
**(*A*) H&E staining (*upper panels*) and MT staining (*lower panels*) of ileal tissues from SAMP1/YitFc mice at 42 weeks of age.** Blue color in MT staining (*arrows*) indicated collagen deposition. (*B*) Ileal histology scores. (*C*) Ileal fibrosis scores. (*D*) Ileal overall disease activities. Prominent ileal fibrosis was found in elafin-overexpressing groups with lentiviral Ctss and Zeb1 overexpression and oral PAR2 agonist GB110 treatment. Ileal fibrosis was ameliorated with lentiviral Ctss and Zeb1 shRNA inhibition and oral PAR2 inhibitor GB88 treatment. Six AKR or SAMP1/YitFc mice per group. Ordinary one-way ANOVA with Tukey tests.

**Figure 10:**
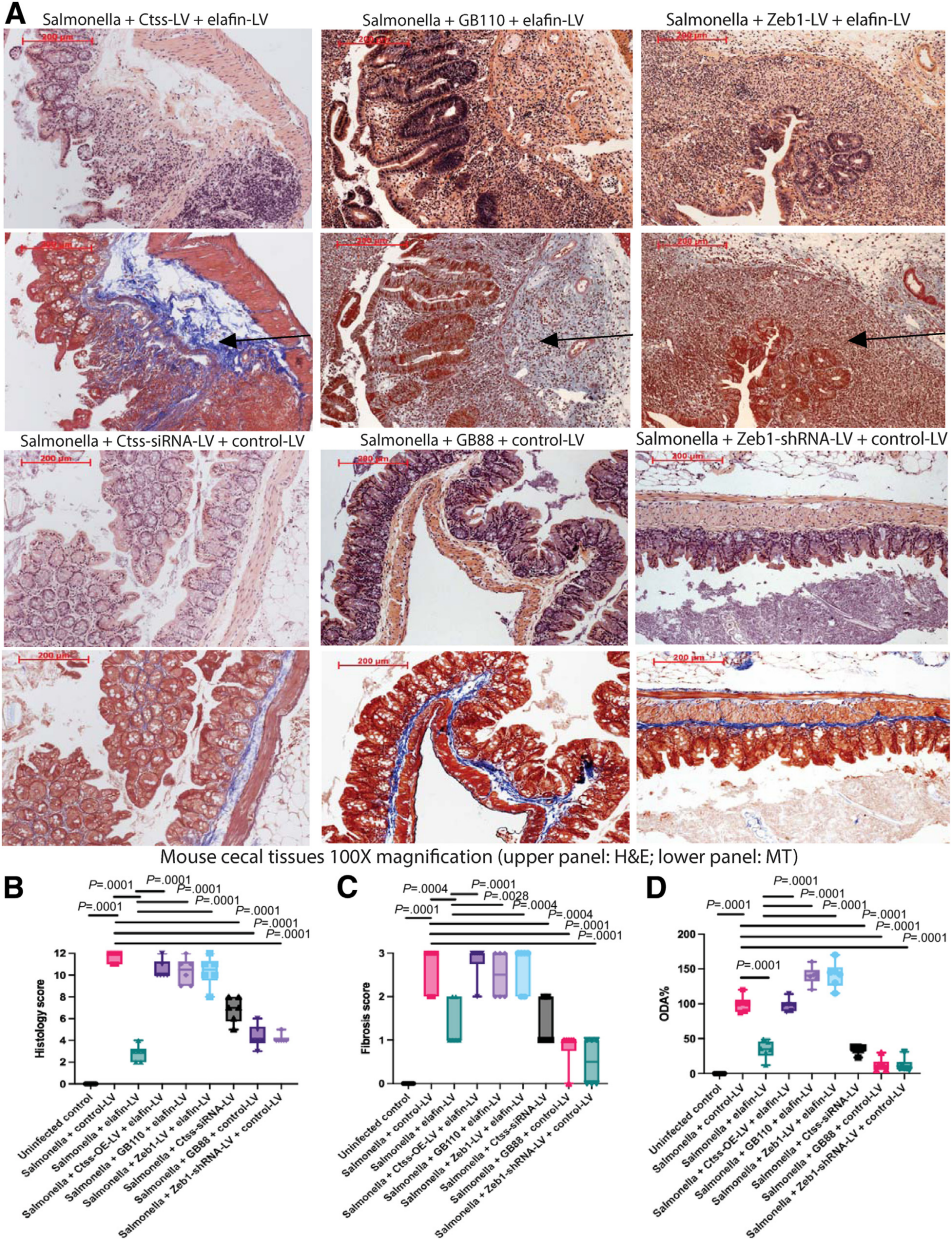
**(*A*) H&E staining (*upper panels*) and Masson Trichrome (MT) staining (*lower panels*) of cecal tissues on day 21.** Blue color in MT staining (*arrows*) indicated collagen deposition in cecal lamina propria of *Salmonella*-infected mice. Prominent cecal fibrosis was found in elafin-overexpressing groups with lentiviral Ctss and Zeb1 overexpression and oral PAR2 agonist GB110 treatment. Cecal fibrosis was ameliorated with lentiviral Ctss and Zeb1 shRNA inhibition and oral PAR2 inhibitor GB88 treatment. (*B*) Cecal histology scores. (*C*) Cecal fibrosis scores. (*D*) Cecal overall disease activities. Six uninfected or *Salmonella*-infected mice per group. Ordinary one-way ANOVA with Tukey tests.

**Figure 11:**
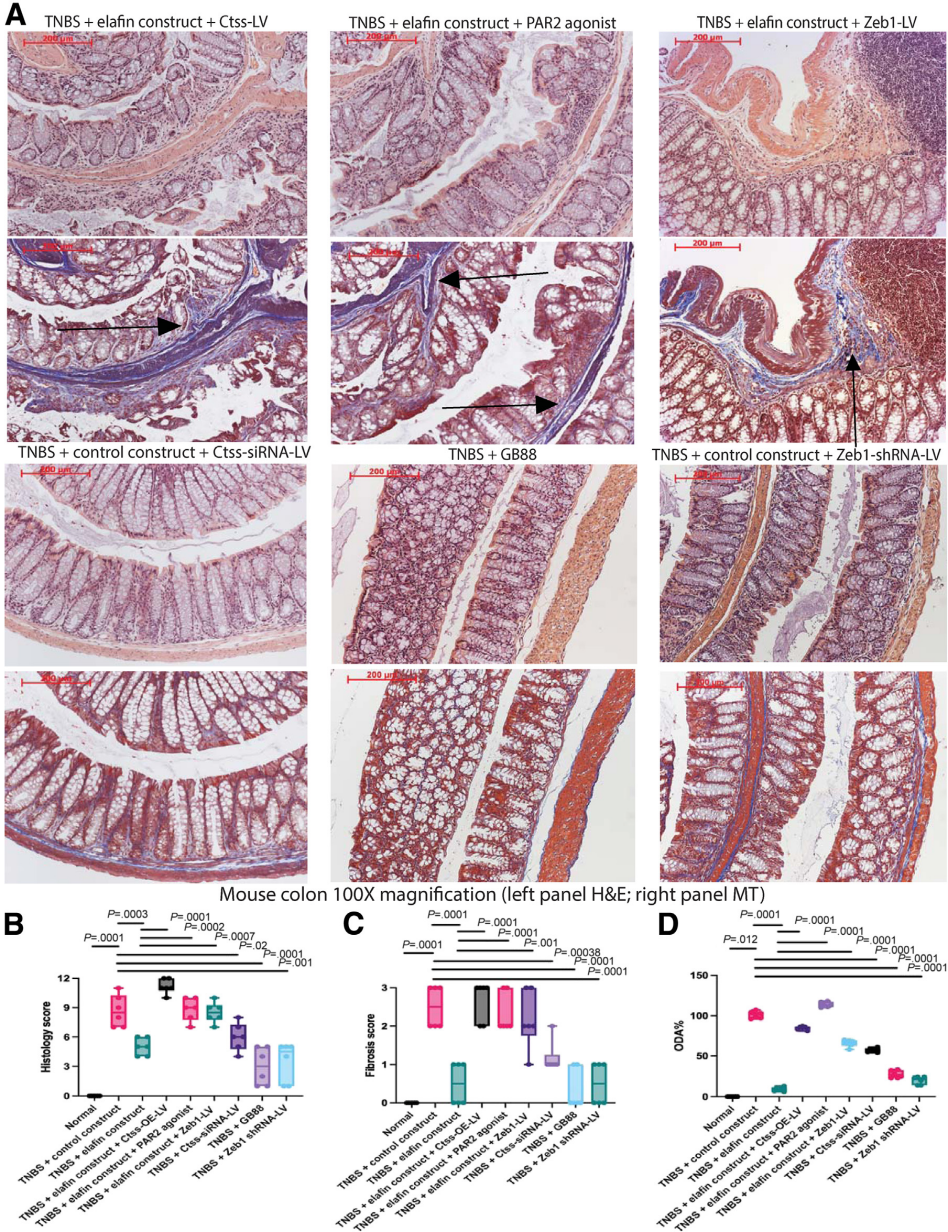
**(*A*) TNBS-treated mice were injected with Ctss-overexpressing lentivirus, Ctss-siRNA lentiviruses, Zeb1-overexpressing lentivirus, or Zeb1-shRNA lentivirus on day 9 after last TNBS injection.** Some mice were injected with 5 mg/kg PAR2 agonist SLIGKV-NH_2_ intracolonically 9, 11, and 13 days after last TNBS injection. GB88 (10 mg/kg/day) was administered via oral gavage. Control or elafin-overexpressing construct was injected intracolonically. H&E staining (*upper panels*) and Masson Trichrome staining (*lower panels*) of colonic tissues are shown. Blue color in MT staining (*arrows*) indicated collagen deposition. (*B*) Colonic histology scores. (*C*) Colonic fibrosis scores. (*D*) Colonic overall disease activities. Prominent colonic fibrosis was found in elafin-overexpressing groups with lentiviral Ctss and Zeb1 overexpression and oral PAR2 agonist GB110 treatment. Colonic fibrosis was ameliorated with lentiviral Ctss and Zeb1 shRNA inhibition and oral PAR2 inhibitor GB88 treatment. Six mice per group. Ordinary one-way ANOVA with Tukey tests.

As we demonstrated the involvement of cathepsin S, PAR2, and ZEB1 in the anti-fibrogenic effect of elafin in intestinal fibroblasts (Figure 3, Figure 4, Figure 5), we further validated their roles in intestinal development in vivo. Inhibition of Ctss, PAR2, and Zeb1 ameliorated ileal fibrosis in SAMP/YitFc mice (Figure 9*A*), cecal fibrosis in *Salmonella*-infected mice (Figure 10*A*), and colonic fibrosis in TNBS-treated mice (Figure 11*A*), with reduced histology scores (Figures 9*B*, 10*B*, and 11*B*), fibrosis scores (Figures 9*C*, 10*C*, and 11*C*), and ODAs (Figures 9*C*, 10*C*, and 11*C*) in all models. Therefore, cathepsin S, PAR2, and ZEB1 regulate intestinal fibrosis development in mice.

### Anti-Fibrogenic Effect of Elafin Is Dependent on Ctss, Protease-Activating Receptor 2, and Zinc Finger E-Box-Binding Homeobox 1 Inhibition in Vivo

We further manipulated cathepsin S, PAR2, and ZEB1 in elafin-overexpressing mice to determine their involvement in the elafin-mediated anti-fibrogenic effect. Lentiviral Ctss and Zeb1 overexpression and PAR2 agonist reversed the anti-fibrogenic effect of elafin overexpression in SAMP1/YitFc mice (Figure 9*A*), *Salmonella*-infected mice (Figure 10*A*), and TNBS-treated mice (Figure 11*A*), with increased histology scores (Figures 9*B*, 10*B*, and 11*B*), fibrosis scores (Figures 9*C*, 10*C*, and 11*C*), and ODAs (Figures 9*C*, 10*C*, and 11*C*) in all models. The efficacies of pharmacologic and molecular manipulations are shown in Table 4. Overall, the anti-fibrogenic effect of elafin overexpression depends on cathepsin S, PAR2, and Zeb1.

**Table 4:** Comparison of Overall Disease Activities and Gene Expression in Mice With Lentiviral and Pharmacologic Manipulations

Lentivirus/drugs	Target	SAMP ileal	Salmonella cecal	TNBS colonic	Overall target mRNA expression or ODA
Ctss-siRNA-LV	ODA	58%	30%	57%	48%
PAR2 inhibitor GB88	ODA	39%	11%	27%	26%
Zeb1-shRNA-LV	ODA	51%	9%	20%	27%
Ctss-siRNA-LV	Ctss	48%	0%	50%	33%
Ctss-siRNA-LV	Zeb1	1%	14%	39%	18%
PAR2 inhibitor GB88	Zeb1	45%	14%	4%	21%
Zeb1-shRNA-LV	Zeb1	18%	19%	0%	12%
Control-LV	ODA	100%	100%	100%	100%
Control-LV + Elafin	ODA	6%	23%	9%	13%
Ctss-LV + Elafin	ODA	211%	97%	85%	131%
GB110/PAR2 agonist	ODA	169%	139%	114%	141%
Zeb1-LV + Elafin	ODA	112%	140%	66%	106%
Control-LV	Ctss	100%	100%	100%	100%
Control-LV + Elafin	Ctss	46%	24%	53%	41%
Ctss-LV + Elafin	Ctss	80%	82%	108%	90%
Control-LV	Zeb1	100%	100%	100%	100%
Control-LV + Elafin	Zeb1	6%	4%	0%	3%
Ctss-LV + Elafin	Zeb1	745%	101%	170%	339%
GB110/PAR2 agonist	Zeb1	444%	223%	140%	269%
Zeb1-LV + Elafin	Zeb1	434%	314%	85%	278%

**Figure 12:**
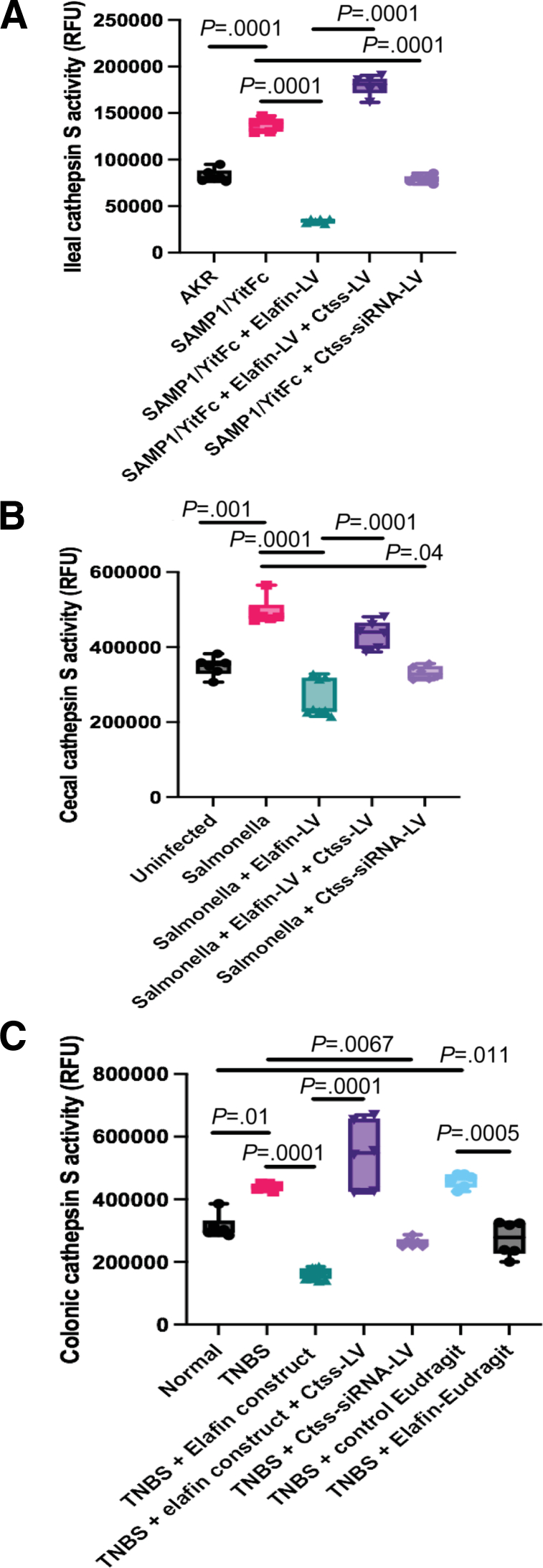
**(*A*) Ileal cathepsin S activities in AKR and SAMP1/YitFc mice.** (*B*) Cecal cathepsin S activities in uninfected and *Salmonella*-infected mice. (*C*) Colonic cathepsin S activities in normal and TNBS-treated mice. Fifty μg tissue lysates in 50 μL CS buffer per reaction. Six mice per group. Ordinary one-way ANOVA with Tukey tests.

Ctss-siRNA lentivirus diminished the intestinal tissue Ctss mRNA expression and cathepsin S activities in fibrotic mice (Table 1, Table 2, Table 3, Table 4, Figure 12*A–C*). Conversely, Ctss-overexpressing lentivirus reversed the elafin-mediated reduction of intestinal tissue Ctss mRNA expression and cathepsin S activities in elafin-overexpressing mice (Table 1, Table 2, Table 3, Table 4, Figure 12*A–C*). Thus, lentiviral manipulation of Ctss expression affected intestinal cathepsin S activity.

**Figure 13:**
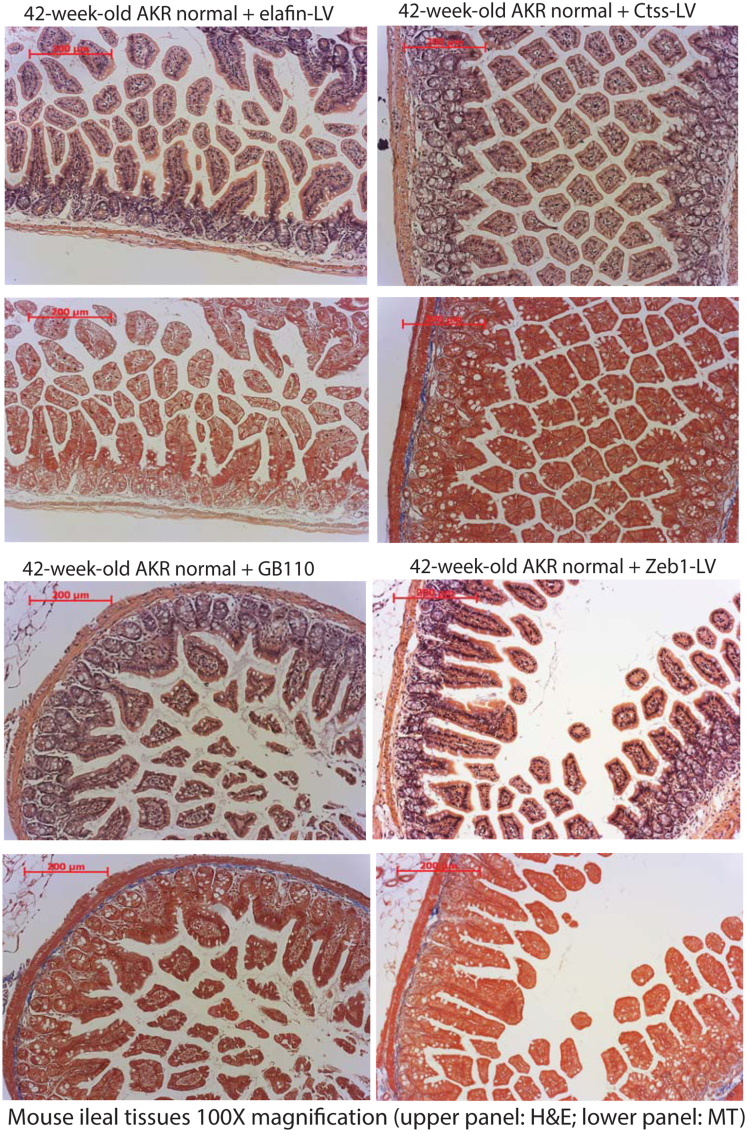
**Elafin-overexpressing lentiviruses, Ctss-overexpressing lentivirus, and Zeb1-overexpressing lentivirus were injected into AKR mice intraperitoneally at 40 weeks of age.** In addition, oral PAR2 agonist GB110 (10 mg/kg/day) was administered via oral gavage from 40 to 42 weeks of age. H&E staining (*upper panels*) and Masson Trichrome (MT) staining (*lower panels*) of ileal tissues did not find histologic injury or fibrosis. Six mice per group.

**Figure 14:**
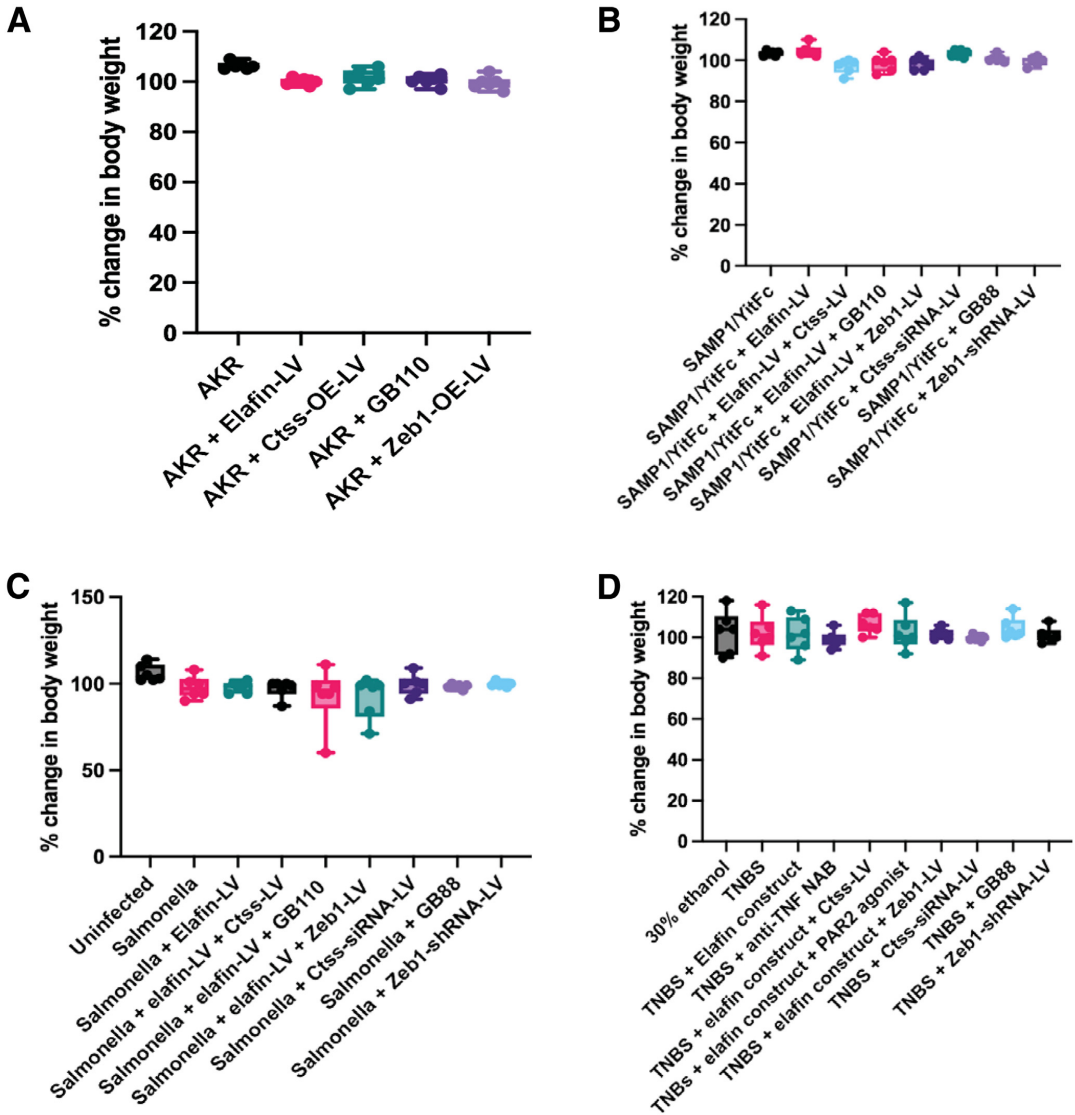
**(*A*) Changes in body weight of AKR mice from 40 to 42 weeks of age.** (*B*) Changes in body weight of SAMP/YitFc mice from 40 to 42 weeks of age. (*C*) Changes in body weight of uninfected and *Salmonella*-infected mice from day 14 to day 21. (*D*) Changes in body weight of normal and TNBS-treated mice from week 5 to week 7. Elafin overexpression and lentiviral/pharmacologic manipulations did not change body weight. n = 6 mice per group.

Lentiviral elafin, Ctss, and Zeb1 overexpression and PAR2 agonist did not affect the normal ileal histology and body weight in control non-fibrotic AKR mice (Figure 13, Figure 14*A*). Similarly, these manipulations did not affect body weight in fibrotic SAMP1/YitFc, *Salmonella*-infected, and TNBS-treated mice (Figure 14*B–D*).

### Oral Elafin-Eudragit-Hydroxypropyl Methylcellulose Formulation Inhibited Colonic Fibrosis in Mice

**Figure 15:**
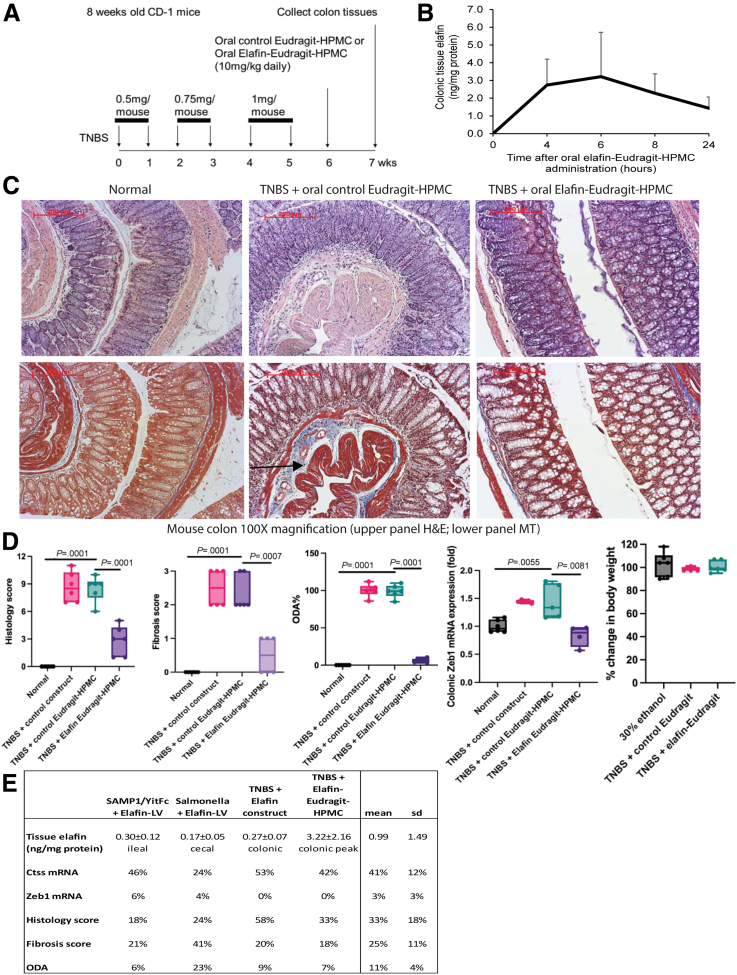
**(*A*) Experimental plan.** The formulation was suspended in mildly acidified (pH 5) water containing 0.5% hydroxypropyl methylcellulose (HPMC) and administered to TNBS-treated mice via oral gavage. The Eudragit polymer releases its drug in the mid-distal ileum and colon under an alkaline environment at ∼pH 8. (*B*) Elafin-Eudragit-HPMC (10 mg/kg) was administered to normal mice via oral gavage. Colonic tissue elafin levels were determined by ELISA (DY1747; R&D Systems). Mean ± standard deviation. (*C*) H&E staining (*upper panels*) and MT staining (*lower panels*) of colonic tissues. Blue color in MT staining (*arrows*) indicated collagen deposition. (*D*) Histology scores, fibrosis scores, overall disease activities, colonic Zeb1 mRNA expression, and changes in body weight. Six mice per group. Ordinary one-way ANOVA with Tukey tests. (*E*) Comparison of intestinal tissue elafin levels and efficiencies in regulating target genes and disease activities. Body weight changes in elafin treatment groups compared with their respective positive control groups. Mean ± standard deviation.

We generated a clinically relevant elafin-Eudragit-hydroxypropyl methylcellulose (HPMC) formulation for oral administration (Figure 15*A*).^22^ Oral elafin-Eudragit-HPMC administration showed peak colonic elafin level at 6 hours and reversed colonic fibrosis with lowered histology and fibrosis scores in TNBS-treated mice (Figure 15*B–D*). Because multiple clinical and endoscopic disease activity scoring systems for IBD suggest the necessity to reduce severe disease activity to 22% to achieve remission (Table 5),^41^ the overall disease activity at 7% reflected remission in the oral formulation-treated group (Figure 15*E*). Both elafin overexpression and elafin-Eudragit-HPMC formulation produced elafin in intestinal tissues (Figure 15*E*), reduced cathepsin S activity (Figure 12*C*), and Ctss and Zeb1 mRNA expression and lowered histology scores, fibrosis scores, and ODAs in fibrotic mice (Figure 15*D* and *E*).

**Table 5:** Comparison of Clinical and Endoscopic Disease Activity Assessment Tools in IBD Patients % of IBD disease activity from severe to remission

Index	CDAI	HBI	PRO-2	St. Mark	Rachmilewitz	Mayo	SES-CD	CDEIS		
Assessment basis	symptom	symptom	symptom	symptom	symptom	both	endoscopic	endoscopic		
Disease	CD	CD	CD	UC	UC	UC	CD	CD		
High remission limit	149	4	7	3	4	2	2	3		
Low severe limit	451	17	35	15	18	11	16	13		
% of disease activity	33	24	20	20	22	18	13	23	Mean 22	Sd 5.82

CDAI, Crohn’s disease activity index; CDEIS, Crohn’s Disease Endoscopic Index of Severity; HBI, Harvey-Bradshaw Index; SES-CD, Simple Endoscopic Score for Crohn’s Disease.

Defining Disease Severity in Inflammatory Bowel Diseases: Current and Future Directions

## Discussion

This report is the first to discover 3 novel targets of intestinal fibrosis (cathepsin S, PAR2, ZEB1). Although the protective role of elafin in mice with colitis was demonstrated previously,^20^^,^^42^ we elucidated a novel anti-fibrogenic mechanism of elafin that involves these 3 targets.

The etiologies of intestinal fibrosis in the 3 mouse models of intestinal fibrosis have not been fully characterized. However, the anti-fibrogenic effects of elafin were robust, as shown by multiple cell and animal approaches. More importantly, elafin inhibited collagen mRNA expression in fresh ileal and colonic tissues from stricturing CD patients (Figures 1*C* and 5*E*), suggesting its potential efficacy against ileal and colonic strictures.

**Figure 16:**
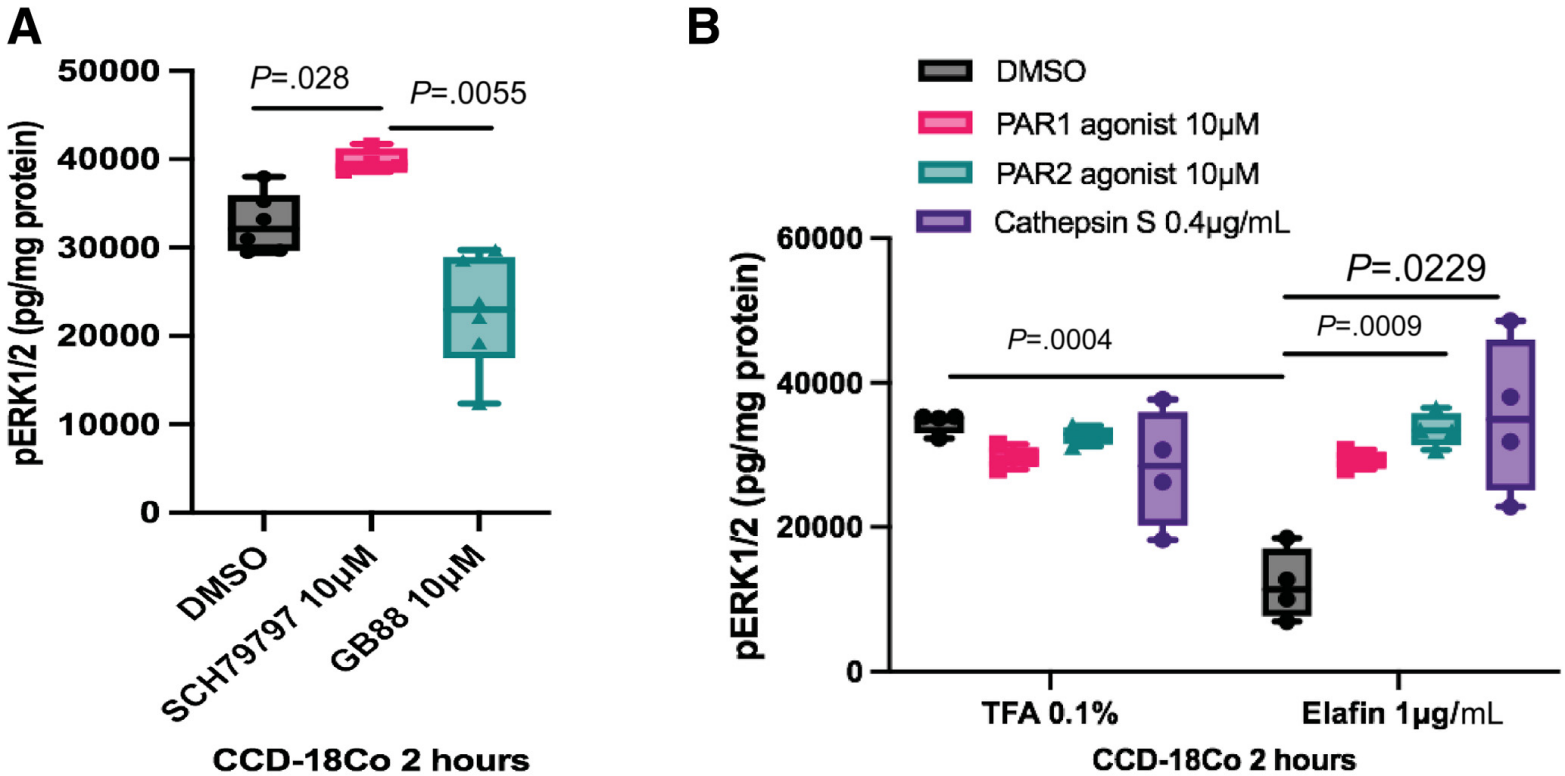
**(*A*) Serum-starved CCD-18Co fibroblasts were treated with DMSO, PAR1 inhibitor (SCH79797), or PAR2 inhibitor (GB88) for 2 hours.** PAR2 inhibitor GB88 inhibited ERK1/2 phosphorylation. Results were pooled from 3 independent experiments. Ordinary one-way ANOVA with Tukey test. (*B*) Serum-starved CCD-18Co fibroblasts were pretreated with DMSO, PAR1 agonist (10 μmol/L), PAR2 agonists (10 μmol/L), or cathepsin S (0.4 μg/mL) for 30 minutes, followed by incubation with elafin (1 μg/mL) for 2 hours. ERK1/2 phosphorylation was determined by ELISA. PAR2 agonist and cathepsin S reversed elafin-mediated inhibition of ERK phosphorylation. Results were pooled from 4 independent experiments. Ordinary one-way ANOVA with Tukey test.

Elafin targets CDSE-induced cathepsin S because the extracellular cathepsin S is associated with the plasma membrane and cleaves near the N-terminus of PAR2,^33^^,^^35^ which activates extracellular signal-regulated kinases.^43^ The elafin-mediated extracellular signal-regulated kinase inactivation reflected diminished cathepsin S and PAR2 activity (Figure 16). Thus, elafin exerts anti-fibrogenic effects by inhibiting cathepsin S and PAR2 activities. Unfortunately, because of limited laboratory capacity, we cannot further characterize the molecular interactions between elafin and cathepsin S.

A previous x-ray crystallography study demonstrated that the primary contact region (from leucine position 20 to leucine position 26) and secondary contact region (from serine position 48 to alanine position 52) of elafin are bound at the active site of porcine pancreatic elastase non-covalently.^44^ A modified elafin with A62D and M63L mutations lost its antiprotease activities without affecting antimicrobial properties.^45^ On the other hand, substitutions of valines at positions 5 and 9 of the elafin amino acid sequence with glycine and glutamines abolished the antiprotease, but not anti-inflammatory, activities of elafin against neutrophil elastase and proteinase 3.^46^ Interestingly, elafin and its closely related secretory leukocyte protease inhibitor (SLPI) possess C-terminal whey acidic protein regions and share similar but non-identical antiprotease, antimicrobial, and anti-inflammatory activities.^47^^,^^48^ Thus, it is difficult to predict the exact region of mature elafin responsible for antiprotease activity against cathepsin S and other proteases.

Although this study discovered cathepsin S as a fibroblast-derived fibrogenic target, microbiota and host cells (epithelial cells and immune cells) can produce other proteases. For example, mast cell tryptase can mediate fibrogenesis in human colonic CCD-18Co fibroblasts.^24^ However, the expression and activity of many proteases and antiproteases in IBD patients can be very complicated. In addition, many of them have multiple targets and functions. This area requires further investigation, but we could not further elucidate their interactions and involvement in CD strictures because of the limitations of assays and samples.

Elafin did not affect T-cell cytokine secretion in CDSE-preconditioned CD-PBMC (Table 6). Elafin also did not affect TNFα secretion in lipopolysaccharide-treated mouse macrophages.^22^ Therefore, the anti-fibrogenic effect of elafin is independent of TNFα, because TNFα neutralization cannot reverse intestinal fibrosis in mice (Figure 8) and CD patients.^4^ We speculate that the reduced Tnf mRNA expression in elafin-overexpressing mice might result from gut barrier protection. Elafin protects the epithelial barrier by inhibiting epithelial elastase 2A hyperactivity independent of PAR2.^19^ Epithelial elastase 2A transgenic mice have colitis with increased colonic Tnf mRNA expression.^19^ The relationship between the intestinal barrier and fibrosis development is unclear and beyond the scope of our study.

**Table 6:** Cytokine Secretion in CD-PBMCs

CD PBMC 6 hours (*pg/mL*)		IFN-gamma mean	SD	IL-10 mean	SD	IL-13 mean	SD	IL-6 mean	SD	IL-7 mean	S	IL-8 mean	SD	TNFα mean	SD
no serum exosome	0.1% TFA	1.94	0.22	4.36	0.96	0.64	0.15	1.86	0.31	3.41	0.08	467.79	1.72	2.86	0.83
No serum exosome	Elafin 1μg/ml	2.69	0.44	4.77	0.42	0.62	0.09	1.85	0.04	3.90	0.08	539.54	58.15	3.58	0.32
CDS serum exosomes	0.1% TFA	2.22	0.40	3.71	0.36	0.82	0.34	1.75	0.05	3.35	0.70	583.98	11.30	3.80	0.21
CDS serum exosomes	Elafin 1μg/ml	3.09	0.37	3.65	0.29	0.84	0.05	1.99	0.14	3.66	0.32	591.44	14.63	4.41	0.40
CDNS serum exosomes	0.1% TFA	2.18	0.31	7.57	1.29	0.43	0.06	1.19	0.02	3.32	0.31	359.15	11.35	2.10	0.54
CDNS serum exosomes	Elafin 1μg/ml	1.64	0.07	7.12	0.63	0.57	0.12	1.04	0.02	2.39	0.35	256.31	20.34	1.59	0.12

NOTE. Results were pooled from 3 CD patients. Ordinary one-way ANOVA test did not find any significant differences.

IFN, interferon; IL, interleukin; PBMC, peripheral blood mononuclear cell; TNF, tumor necrosis factor.

Although elafin and SLPI possess antiprotease, anti-inflammatory, and antimicrobial properties,^49^ SLPI promotes cancer metastasis and is unsuitable for therapeutic development.^50^ Elafin should not affect colorectal cancer risk because colonic elafin expression is not associated with the type, stage, and locations of the colorectal tumors or the survival of the patients (COADREAD database). Compared with non-IBD patients, CD patients do not have altered intestinal SLPI mRNA and protein expression.^17^ SLPI deficiency does not affect bleomycin-induced lung fibrosis development in mice.^51^ We speculate that SLPI is unlikely to exert anti-fibrogenic activities in activated fibroblasts because cathepsin S can cleave and inactivate SLPI.^52^

Eudragit-FS30D-HPMC polymer protects the therapeutic agent through the stomach and releases it in humans’ mid-distal ileum and colon.^53^ For example, Asacol is a Eudragit-coated mesalamine for treating IBD. Like elafin-overexpressing bacteria,^20^^,^^42^ the elafin-Eudragit formulation maximized elafin delivery to the diseased intestine (Figure 15*E*). Both lentiviral elafin overexpression and elafin-Eudragit formulation increased circulating elafin levels in mice.^54^ However, systemic delivery of elafin is unlikely to be useful for clinical applications because elafin has a short half-life in circulation because of rapid renal elimination.^55^ IBD patients, including stricturing CD patients, have increased elafin expression in mesenteric fat and elevated circulating elafin levels.^6^ Therefore, delivery of elafin to the fibrotic intestines should precisely confer anti-fibrogenic effects regardless of circulating elafin levels.

Direct exposure to elafin did not induce collagen mRNA expression in fresh human colonic tissues (Figure 1*C*), whereas lentiviral elafin overexpression did not cause ileal fibrosis in normal AKR mice (Figure 13). Therefore, we believe that elafin formulation delivery cannot initiate or promote intestinal fibrosis because intestinal fibrosis is a multifactorial process.^1^

Intestinal strictures are classified into inflammatory, fibrotic, and mixed phenotypes.^56^ Anti-inflammatory drugs can diminish inflammatory strictures, but fibrotic strictures have no known anti-fibrogenic drugs. Imaging analysis is inaccurate in differentiating stricture phenotypes, whereas ileocolonoscopy may not access the strictures for evaluation, especially in sites with multiple strictures.^56^ It is unfeasible to define phenotype-based therapy in current clinical practice because most CD patients’ phenotypes are unknown. We believe that the elafin-Eudragit FS30D formulation can cover intestinal inflammation and fibrosis because the same formulation inhibited obesity and hyperglycemia in high-fat diet–treated mice,^22^ which had low-grade chronic intestinal inflammation.^57^

In summary, elafin inhibits cathepsin S-dependent PAR2 activity and reduces ZEB1 and collagen expression in intestinal fibroblasts. The significance of this study is to gain insight into the mechanism of intestinal fibrosis and discover a potential anti-fibrogenic approach.

## Methods

### Frozen Human Colonic Tissues

Frozen colonic tissue samples of non-IBD, UC, and CD patients were collected from the Cedars-Sinai Medical Center during the surgical resection of diseased tissues from 2010 to 2014 prospectively and cryopreserved until the study.^40^ The Cedars-Sinai Institutional Review Board (#3358 and #23705) and UCLA Institutional Review Board (11-001527) approved the study. Informed consent was obtained from all subjects by the Cedars-Sinai Medical Center. UCLA Institutional Review Board waived separate informed consent. Frozen human colonic tissues were used for comparing gene expression in non-IBD, UC, stricturing CD, and non-stricturing CD patients.^6^

### Human Serum Samples

Serum samples of normal, UC, and CD patients were prospectively collected from UCLA from 2012 to 2015. The physicians requested the medically indicated blood collection. UCLA Institutional Review Board (IRB 12-001499) approved this study. Separate informed consent was waived by UCLA IRB because UCLA Pathology obtained written informed consent from all subjects. The pooled sera from 12 stricturing CD patients were used for preparing serum exosomes (CDSE).^6^

Serum exosomes were prepared by total exosome isolation reagent (#4478360; Thermo Fisher Scientific, Waltham, MA). In short, the serum sample (1 mL) was centrifuged at 2000*g* for 30 minutes to remove cells and debris. The supernatant was then mixed with 200 μL of total exosome isolation reagent and refrigerated at 4^o^C for 30 minutes. The mixture was then centrifuged at 10,000*g* for 10 minutes at room temperature. After removing the supernatant, the pellet was resuspended with 250 μL phosphate-buffered saline. The protein concentration in the serum exosomes was quantified by bicinchoninic acid protein assay (#23225; Thermo Fisher Scientific).

### Fresh Human Intestinal Tissues

Fresh colonic tissues from colon cancer patients with normal histology and ileal tissues from stricturing CD patients with fibrotic morphology were obtained from UCLA Surgical Pathology from 2020 to 2021. UCLA IRB (12-001499) approved the study. Fresh human intestinal tissues were cut into 3 × 3 mm and incubated in serum-free RPMI1640 medium with or without 100 μg/mL CDSE. Two hours later, elafin (1 μg/mL) was added and incubated for 24 hours. Fresh human intestinal tissues were used for assessing elafin-mediated effects.

Baseline characteristics of all intestinal tissues and serum samples are shown in Table 7.

**Table 7:** Baseline Characteristics of Frozen Human Colonic Tissue Samples, Fresh Colonic Tissue Samples From Colon Cancer Patients, Fresh Ileal Tissue Samples From Stricturing CD Patients, Primary Stricturing CD Patient-Derived Intestinal Fibroblasts, Primary Peripheral Blood Mononuclear Cells From CD Patients, and Human Serum Samples

Colonic tissues (mean ± SD)	Non-IBD	UC	CD without stricture	CD with stricture
Elafin mRNA expression (fold)	5.7 ± 1.9	11.8 ± 2.7	5.4 ± 1	2.9 ± 2.3
Age at collection (*y*)	62 ± 13.9	43 ± 15.1	45 ± 19	36 ± 23.6
Gender (% male)	73	55	62	50
Histology score	2.6 ± 1.9	7.5 ± 2.9	8.8 ± 3.7	8.6 ± 4.6
Simple colitis activity score	N/A	6.8	N/A	N/A
Harvey Bradshaw Index	N/A	N/A	7.5 ± 1.2	5 ± 1.9
% of biologics	0	24	50	40
% of 6-mercaptopurine or steroid	0	51	66	33
Duration of disease (*y*)	26 ± 3	12 ± 2	8 ± 3	18 ± 3
n	40	52	28	15

Serum samples (mean ± SD)	Non-IBD	UC	CD without stricture	CD with stricture
Serum elafin levels (*pg/mL*)	7939 ± 791	12,987 ± 1124	7042 ± 520	11,263 ± 1818
Age at collection (*y*)	46 ± 12	40 ± 10	34 ± 11	40 ± 13
Gender (% male)	42	35	40	60
Harvey Bradshaw Index	N/A	N/A	3.7	5
Partial Mayo score	N/A	2.1 ± 0.4	N/A	N/A
% of biologics	N/A	13	40	46
% of 6-mercaptopurine or steroid	N/A	12	33	42
Duration of disease (*y*)	N/A	6 ± 2	11 ± 1.4	11 ± 2.3
n	12	23	33	20

Fresh colonic tissues from colon cancer						
Patient	1	2	3	4	5	6
Age (*y*)	63	58	73	38	68	45
Gender	Female	Male	Female	Male	Male	Male
		Moderately	Moderately		Adenocarcinoma	Adenocarcinoma
		invasive	differentiated		of descending	of sigmoid
Disease	Adenocarcinoma	adenocarcinoma	adenocarcinoma	Diverticulitis	colon	colon
		Proximal				
Disease location	Ascending colon	ascending colon	Ascending colon	Sigmoid colon	Descending colon	Sigmoid colon

CD-HIF	Fresh ileal tissues from CD patients							
Patient	1	2	3	4	Patient	1	2	3
Age (*y*)	41	45	66	68	Age (*y*)	41	45	48
Gender	Male	Female	Male	Female	Gender	Male	Female	Male
							CD	
Disease	CD stricture	CD stricture	CD stricture	CD stricture	Disease	CD stricture	stricture	CD stricture
Disease location	Ileum	Ileum	Colon	Colon	Disease location	Ileum	Ileum	Ileum
					Medication	Budesonide	Stelara	Humira

Stemcell Technologies CD PBMC 70052			
Lot	200871901C	1010113306	200971001C
Age (*y*)	48	25	32
Sex	Female	Female	Male
Ethnicity	Caucasian African American		Caucasian
Diagnosis date	42369	38353	40908
Process date	44062	42350	44084
Smoker	No	No	No
		VSL#3, Tri-	
		Previfem,	
Medications	Vitamin D	Tamiflu	None

For inclusion criteria, IBD, intestinal strictures, and colon cancer were diagnosed by gastroenterologists as described previously.^6^ For exclusion criteria, pregnant women, prisoners, minors younger than age 18, concurrent acute infection (cytomegalovirus infection, *Clostridium difficile* infection*,* and tuberculosis), and malignant conditions were excluded.

### Intestinal Fibroblast and Epithelial Cell Culture

CD-HIFs were prepared from intestinal tissues in stricturing CD patients.^6^ Baseline characteristics of the patients are shown in Table 7. In short, the intestinal mucosa was stripped from submucosa and muscularis propria and cut into 1 × 1 mm pieces. The mucosal tissues were washed with phosphate-buffered saline and then digested with 1 mg/mL collagenase II, 0.3 mg/ml DNase I, and 2 mg/mL hyaluronidase at 37^o^C for 30 minutes with shaking. Next, the dissociated cells were cleared through a 40-μm cell strainer and centrifuged at 10,000*g* for 5 minutes. After removing supernatants, the cell pellets were suspended with fibroblast medium (M2267; Cell Biologics, Chicago, IL) and cultured on gelatin-coated culture flasks. We cultured primary fibroblasts during passages 3–10 for experiments. Serum-starved CD-HIF were pretreated with 100 μg/mL CDSE for 2 hours to mimic the CD environment and induce fibrogenesis.^6^

Human colonic CCD-18Co fibroblasts (CRL-1459, ATCC) were cultured in minimal Eagle medium with 10% fetal bovine serum and 1% penicillin-streptomycin.^10^^,^^40^ All cells were grown to 80% confluence and then switched to serum-free medium overnight for experiments. Serum-starved fibroblasts were pretreated with either 0.1% trifluoroacetic acid as a vehicle or 10 ng/m: TGF-β1 for 2 hours, followed by incubation with elafin (#AS-61641; AnaSpec, Fremont, CA) for 2–24 hours. Details of other chemicals used in this study are shown in Table 8.

**Table 8:** Catalog and Batch Numbers of Reagents

	Catalog number	Lot number
Human PCR assays	Source: Thermo Fisher	
COL1A2	(Hs01028956_m1)	1811559
COL3A1	(Hs00943809_m1)	1771975
TGF-β1	(Hs00998133_m1)	1543822
N-cadherin	(Hs00983056_m1)	1248923
ZEB1	(Hs01566408_m1)	2005137
CTSS	(Hs00175407_m1)	1929800
18S (endogenous control)	(Hs99999901_s1)	1739902
Mouse PCR assays	Source: Thermo Fisher	
Col1a2	(Mm00483888_m1)	1782343
Col3a1	(Mm00802300_m1)	1763098
Tgf-β1	(Mm01178820_m1)	1313258
Zeb1	(Mm00495564_m1)	1868064
Vim	(Mm01333430_m1)	1910567
Acta2	(Mm00725415_s1)	2021653
Tnf	(Mm00443258_m1)	1980751
Emr1/Adgre1	(Mm00802529_m1)	1788225
Ctss	(Mm01255859_m1)	1883639
Gapdh (endogenous control)	(Mm99999915_g1)	1927287

Elafin-fibrosis project Elafin	Vendor Anaspec	Catalog # AS-61641	Purity 95%	Batch 2055773
Protease-activated receptor-2, amide SLIGKV-NH2	MCE	HY-P0283	98.33%	29492
2-Furoyl-LIGRLO-amide	MCE	HY-P1314	99.87%	57931
GB-110 hydrochloride	MCE	HY-120528A	99.94%	50389
GB-88	Eton Bioscience	2300100052	98.10%	1416435-96-5
SCH79797 hydrochloride	Sigma	SML1939	98%	57328
Control lentivirus Elafin overexpressing Lentivirus Mouse Zeb1 shRNA lentivirus Mouse Zeb1 overexpressing lentivirus Mouse Ctss siRNA lentivirus	OriGene OriGene OriGene OriGene ABMgood	PS1000064V RC203136L1V TL513177V MR223095L2V 171210940296	N/A N/A N/A N/A N/A	133EE26 SR156301 SR168040 SR180321 V21H18I
Mouse Ctss overexpressing lentivirus Control construct Human elafin overexpressing Construct Human ZEB1 overexpressing construct	ABMgood OriGene OriGene Origene	171210640196 PS100001 RC203136 RC217704	N/A N/A N/A N/A	V21H25I N/A N/A BJ10116-194610948

Primary human colonic epithelial cells (H6047; Cell Biologics) were cultured in epithelial cell medium (H6621; Cell Biologics) until 90% confluence. Then, the cells were switched to serum-free medium overnight for TGF-β1, CDSE, and elafin treatment.

At the end of the experiments, the cells were lysed with radioimmunoprecipitation assay buffer (#89900; Thermo Fisher Scientific) containing protease and phosphatase inhibitor cocktail (PPC1010; Sigma-Aldrich, St Louis, MO) for ELISA. We used ELISA to measure protein levels of ProCOL1A1 (DY6220-05; R&D Systems, Minneapolis, MN), ERK1/2 phosphorylation (DYC1018B; R&D Systems), ZEB1 (MBS774017; MyBioSource, San Diego, CA), and COL1A2 (MBS2701496; MyBioSource) in cell lysates. Alternatively, the cells were lysed with Qiagen’s RLT buffer for RNA experiments.

### Protease Array

Serum-starved CD-HIF (1 × 10^7^ cells/well) were treated with 100 μg/mL CDSE for 2 hours. Serum-starved CCD-18Co colonic fibroblasts (1 × 10^7^ cells/well) were treated with 10 ng/mL TGF-β1 for 2 hours. Conditioned media 500 μL were loaded to Proteome Profiler Human Protease Array membranes (ARY021B; R&D Systems) and incubated with detection antibody overnight at 4^o^ C with shaking. The membranes were then washed and incubated with streptavidin-horseradish peroxidase and substrate. A Bio-Rad ChemiDoc Imaging system (Hercules, CA) captured the luminescence signals emitted from the membranes and generated the images for analysis. Bio-Rad Image software quantified the signal intensities of individual proteases and references in images. We used Excel (Microsoft, Redmond, WA) to calculate the ratios of individual protease signals over reference signals.

### Cathepsin S Activity Measurement

We used a fluorescence-based activity assay kit (ab65307; Abcam, Cambridge, UK) to measure the cathepsin S activity. This assay kit uses the preferred cathepsin-S substrate sequence VVR labeled with AFC (amino-4-trifluoromethyl coumarin). Cell lysates or other samples that contain cathepsin S will cleave the synthetic substrate VVR-AFC to release free AFC. The released AFC can be quantified using a fluorometer or fluorescence plate reader.

We performed the cathepsin S activity assays in cell-free conditions to determine the direct interactions between purified elafin and cathepsin S proteins without interference from other cell components. Elafin (0.5–10 μg/mL final concentration), cathepsin S (0.4 μg/mL final concentration), and CS inhibitor (20 μmol/L final concentration, provided by the assay kit) were added to a mixture of CS reaction buffer and CS substrate buffer (200 μmol/L final concentration).

To measure cathepsin S activities in cells and tissues, cell culture media, cell lysates, and tissue lysates were first centrifuged for 10,000*g* at 4^o^C for 10 minutes and filtered through 40 μm to remove debris. The filtered supernatants were then added to a mixture of CS reaction buffer and CS substrate buffer (200 μmol/L final concentration).

The 100 μL/well mixture was incubated at 37^o^C for 1 hour. Relative fluorescence units were read with 400 nm excitation and 505 nm emission in clear-bottom dark-wall 96-well plates. Specific changes in protocols are mentioned in figure legends.

### Crohn’s Disease Patient-Derived Peripheral Blood Mononuclear Cells

CD-PBMCs from 3 CD patients (#70052; STEMCELL Technologies, Vancouver, Canada) were cultured in RPMI1640 medium containing 10% exosome-depleted fetal bovine serum (A2720803; Thermo Fisher Scientific) and 1% penicillin-streptomycin. Baseline characteristics are shown in Table 7. CD-PBMCs were preconditioned with 100 μg/mL stricturing CD or non-stricturing CDSE for 2 hours and then exposed to either 0.1% TFA or 1 μg/mL elafin for 6 hours. The cells were removed by centrifugation at 10,000*g* for 5 minutes at 4^o^ C. The cell supernatants were collected for a 13-plex cytokine multiplex assay (HSTCMAG28SPMX13; Millipore Sigma, St Louis, MO).

### Animal Experiments

All animal studies were approved by UCLA Institutional Animal Research Committee (#2007-116). All methods were compliant with the ARRIVE guidelines. Mice were randomized and assigned to cages by animal facility staff in a blinded manner and housed in the UCLA animal facility under standard environmental conditions. All interventions were performed during the light cycle.

#### SAMP1YitFc

We used 40-week-old male and female SAMP1/YitFc mice (#009355; Jackson Laboratories, Bar Harbor, ME). This model develops chronic ileitis with preexisting ileal fibrosis around 40 weeks of age. We used 40-week-old AKR mice (#000648; Jackson Laboratories) as a parental normal control strain.^58^

Control lentivirus (PS100064V; OriGene, Rockville, MD), elafin-overexpressing lentiviruses (RC203136L1V; OriGene), Ctss-overexpressing lentivirus (#171210640196; Applied Biological Materials, Richmond, BC, Canada), Ctss-siRNA lentivirus (#171210940296; Applied Biological Materials), Zeb1-shRNA lentivirus (TL513177V; OriGene), and Zeb1-overexpressing lentivirus (MR223095L2V; OriGene) were injected to AKR or SAMP1/YitFc mice intraperitoneally once at 40 weeks of age. In addition, PAR2 agonist GB110 (HY-120528A; MedChemExpress [MCE], Monmouth Junction, NJ) or PAR2 inhibitor GB88 was given via oral gavage from 40 to 42 weeks of age. Ileal tissues were collected for analyses at 42 weeks of age.

Because SAMP1/YifFc mice were inefficient breeders and had low availability, we conducted the experiments in batches of 4 mice per batch. The SAMP1/YitFc mice were kept in cohousing during weeks 8–40 and then assigned to various groups in single housing conditions during weeks 40–42. We conducted 12 rounds of experiments in total.

#### Salmonella

We first treated 8-week-old male and female 129Sv/J mice (000691; Jackson Laboratories) with 20 mg streptomycin via oral gavage. The mice then received *Salmonella typhimurium* SL1344 strain (10^8^ cfu) by oral gavage 1 day later. We used uninfected 8-week-old 129Sv/J mice for the control group and kept them along with the infection course. Cecal fibrosis develops from day 14 to day 21 after infection.^39^

Control lentivirus (PS100064V; OriGene), elafin-overexpressing lentiviruses (RC203136L1V; OriGene), Ctss-overexpressing lentivirus (#171210640196, Applied Biological Materials), Ctss-siRNA lentivirus (#171210940296; Applied Biological Materials), Zeb1-shRNA lentivirus (TL513177V; OriGene), and Zeb1-overexpressing lentivirus (MR223095L2V; OriGene) were injected into the infected mice intraperitoneally once on day 14 after infection. In addition, PAR2 agonist GB110 (HY-120528A; MCE) or PAR2 inhibitor GB88 was given daily via oral gavages from day 14 to day 21 after infection. Cecal tissues were collected for analyses on day 21 after infection.

All *Salmonella* infection experiments were conducted in 3 rounds with 3 male and 3 female mice. Mice were kept in cohousing throughout days 0–21 in the first 2 rounds. Because some mice died during the first 2 rounds of experiments, we conducted the third round to compensate for the loss of mice. Mice in the third round were kept in cohousing during days 0–14 and then single housing during days 14 and 21.

#### TNBS

We injected 8-week-old male and female outbred CD-1 mice (#022; Charles River Laboratories, Wilmington, MA) with TNBS solution in 30% ethanol via enema weekly 6 times (weeks 0, 1, 2, 3, 4, and 5). Thirty percent ethanol was used to help the penetration of TNBS into the colonic mucosa. Colonic fibrosis typically develops 1 week after the last injection (week 6). The normal control group was treated with 6 weekly injections of 30% ethanol via enema.

Some mice received a single intracolonic injection of 5 μg/mouse of either control construct (PS100001) or elafin-overexpressing construct (RC203136) from OriGene via InvivoJetPEI transfection reagent (201-10G; Polyplus, Illkirch-Graffenstaden, France) on the ninth day after the last TNBS injection. In addition, anti-TNFα neutralizing antibodies (BE0058; BioXCell, Lebanon, NH) were injected intraperitoneally.

TNBS-treated mice were injected with Ctss-overexpressing lentivirus (#171210640196) or Ctss-siRNA lentiviruses (#171210940296; Applied Biological Materials, Inc), Zeb1-overexpressing lentivirus (MR223095L2V), or Zeb1-shRNA lentivirus (TL513177V; OriGene) on the ninth day after the last TNBS injection. In addition, some mice were injected with 5 mg/kg PAR2 agonist SLIGKV-NH_2_ (HY-P0283; MCE) intracolonically on days 9, 11, and 13 after the last TNBS injection. PAR2 inhibitor GB88 (10 mg/kg/day) was administered via oral gavage. Colonic tissues were collected 2 weeks after the last TNBS injection.

All TNBS colitis experiments were conducted in 2 rounds, with 3 male mice per group in the first round and 3 female mice per group in the second round. Mice were kept in cohousing condition throughout weeks 1–7.

### Production of Elafin-Eudragit-Hydroxypropyl Methylcellulose

In Texas, Southwest Research Institute produced the oral elafin-Eudragit-FS30D formulation via a material transfer agreement (MTA2019-00000337). First, elafin was coated with Eudragit FS30D polymer. This pH-responsive polymer is insoluble in acid but dissolves in a mildly alkaline environment (ie, pH 7 or above), which is optimal for colonic delivery. Next, elafin-Eudragit was packaged into microparticles using a Southwest Research Institute–patented spinning disk atomization technology. This packaging technology prevented leakage of elafin in acidic conditions. Finally, the elafin-Eudragit microparticles were dried. The resulting powder was suspended in 0.5% HPMC in water for oral gavage.

### Histologic Evaluations

Intestinal tissue injury was evaluated with H&E staining, whereas extracellular matrix deposition was identified by Masson Trichrome staining. H&E- and Masson Trichrome–stained microphotographs were recorded at multiple locations and scored by 2 investigators blindly.^10^^,^^40^ The chronic intestinal injury was scored in terms of mucosal transformation (0/3/6), round cell infiltration in the lamina propria mucosa (0-3), goblet cell death (0/1), tela submucosa fibrosa (0/1), and granuloma (0/1). These parameters result in a total score (0–12).^59^ In addition, intestinal fibrosis was scored on a scale of normal, mild, moderate, and severe (0–3).^39^

### Quantitative Real-Time Reverse Transcription Polymerase Chain Reaction

Total RNA was isolated by an RNeasy kit (#74104; Qiagen, Hilden, Germany) and reverse transcribed into cDNA (#4368813; Thermo Fisher Scientific). Polymerase chain reactions were run with cDNA, iTaq Universal SYBR Green Supermix (1725120; Bio-Rad), and TaqMan real-time polymerase chain reaction assays (Table 8) in a Bio-Rad CFX384 system.^60^ After normalization with endogenous control genes, relative mRNA quantification was performed by comparing test and control groups. The fold changes are expressed as 2^ΔΔCt^. Fold-change values greater than 1 indicate a positive or an up-regulation. Conversely, fold-change values less than 1 indicate a negative or down-regulation.

### Calculation of Overall Disease Activities

We converted histology scores, fibrosis scores, and intestinal gene expression into percentages to help compare between groups and models of intestinal fibrosis. It included several fibrosis-related and inflammation-related genes commonly reported by our and other intestinal fibrosis research groups. *Col1a2* and *Col3a1* are fibrosis signatures.^26^^,^^27^^,^^61^^,^^62^ *Col3a1* was excluded in the *Salmonella* model because *Salmonella* infection did not affect cecal *Col3a1* mRNA expression.^27^ *Acta2*, *Vim*, and *Zeb1* are fibroblasts and epithelial-mesenchymal transition markers.^38^^,^^63^^,^^64^ *Tnf* and *Emr1* are inflammation markers.^40^^,^^65^ The mRNA expression lower than the control group in negative percentage values was assigned 0, indicating complete inhibition. ODA is the average value of these parameters.

### Power Analysis

The colonic tissue cohort of 40 non-IBD, 52 UC, and 43 CD patients provided adequate power to detect colonic elafin mRNA expression.^6^ At least 3 mice per group were required to achieve a statistically significant difference in histology score between the TNBS group (8.33) and TNBS + anti-TNFα (6.4) group with standard deviation = 0.93, alpha = 0.5, and power = 0.8. Our study with 6 mice per group satisfied this requirement. We did not perform power analysis for in vitro experiments but followed the common practice of performing in vitro experiments 3 times or more independently.

### Statistical Analysis

Results were expressed as mean ± standard deviation. We used Graphpad Prism 9 (San Diego, CA) to perform multiple-group comparisons using ordinary one-way analyses of variance (ANOVAs) with Tukey post hoc tests and two-group comparisons using Student *t* tests. The *P* values of statistical significance are shown in each figure or table.
